# Most *Sinorhizobium meliloti* Extracytoplasmic Function Sigma Factors Control Accessory Functions

**DOI:** 10.1128/mSphereDirect.00454-18

**Published:** 2018-10-10

**Authors:** Claus Lang, Melanie J. Barnett, Robert F. Fisher, Lucinda S. Smith, Michelle E. Diodati, Sharon R. Long

**Affiliations:** a Department of Biology, Stanford University, Stanford, California, USA; University of Iowa; Institut National de la Recherche Agronomique, France; Penn State University; ETH Zurich, Institute of Microbiology

**Keywords:** *Rhizobium*, *Sinorhizobium*, microarrays, sigma factors, symbiosis

## Abstract

Bacteria must sense alterations in their environment and respond with changes in function and/or structure in order to cope. Extracytoplasmic function sigma factors (ECF σs) modulate transcription in response to cellular and environmental signals. The symbiotic nitrogen-fixing alphaproteobacterium Sinorhizobium meliloti carries genes for 11 ECF-like σs (RpoE1 to -E10 and FecI). We hypothesized that some of these play a role in mediating the interaction between the bacterium and its plant symbiotic partner. The bacterium senses changes in its immediate environment as it establishes contact with the plant root, initiates invasion of the plant as the root nodule is formed, traverses several root cell layers, and enters plant cortical cells via endocytosis. We used genetics, transcriptomics, and functionality to characterize the entire S. meliloti cohort of ECF σs. We discovered new targets for individual σs, confirmed others by overexpressing individual ECF σs, and identified or confirmed putative promoter motifs for nine of them. We constructed precise deletions of each ECF σ gene and its demonstrated or putative anti-σ gene and also a strain in which all 11 ECF σ and anti-σ genes were deleted. This all-ECF σ deletion strain showed no major defects in free-living growth, in Biolog Phenotype MicroArray assays, or in response to multiple stresses. None of the ECF σs were required for symbiosis on the host plants Medicago sativa and Medicago truncatula: the strain deleted for all ECF σ and anti-σ genes was symbiotically normal.

**IMPORTANCE** Fixed (reduced) soil nitrogen plays a critical role in soil fertility and successful food growth. Much soil fertility relies on symbiotic nitrogen fixation: the bacterial partner infects the host plant roots and reduces atmospheric dinitrogen in exchange for host metabolic fuel, a process that involves complex interactions between the partners mediated by changes in gene expression in each partner. Here we test the roles of a family of 11 extracytoplasmic function (ECF) gene regulatory proteins (sigma factors [σs]) that interact with RNA polymerase to determine if they play a significant role in establishing a nitrogen-fixing symbiosis or in responding to various stresses, including cell envelope stress. We discovered that symbiotic nitrogen fixation occurs even when all 11 of these regulatory genes are deleted, that most ECF sigma factors control accessory functions, and that none of the ECF sigma factors are required to survive envelope stress.

## Abstract (precis)

Fixed (reduced) soil nitrogen plays a critical role in soil fertility and successful food growth. Much soil fertility relies on symbiotic nitrogen fixation: the bacterial partner infects the host plant roots and reduces atmospheric dinitrogen in exchange for host metabolic fuel, a process that involves complex interactions between the partners mediated by changes in gene expression in each partner. Here we test the roles of a family of 11 extracytoplasmic function (ECF) gene regulatory proteins (sigma factors [σs]) that interact with RNA polymerase to determine if they play a significant role in establishing a nitrogen-fixing symbiosis or in responding to various stresses, including cell envelope stress. We discovered that symbiotic nitrogen fixation occurs even when all 11 of these regulatory genes are deleted, that most ECF sigma factors control accessory functions, and that none of the ECF sigma factors are required to survive envelope stress.

## INTRODUCTION

Sinorhizobium meliloti, a Gram-negative alphaproteobacterium, can live as a heterotrophic soil saprophyte or in symbiosis with a host plant such as Medicago sativa or Medicago truncatula (1, 2). Symbiosis proceeds by stages as the bacteria stimulate the plant root to form nodules, invade via an infection thread across multiple cell layers, and infect plant cells in the nodule interior (3). The endosymbiotic bacteria differentiate into bacteroids to fix nitrogen, providing it to the plant in exchange for carbohydrate fuel (4, 5). As S. meliloti transits from soil to nodule, it encounters a succession of new environments and must respond accordingly.

Transcriptional regulation is a key feature of S. meliloti adaptation to the plant environment (6). Plant flavonoids stimulate the bacterial transcription factor NodD to induce expression of the bacterial nodulation (*nod*) genes (7, 8), which encode enzymes that synthesize Nod factor, which provokes formation of root nodules (1). Another key transcriptional regulator is the FixL-FixJ two-component system, which induces the expression of the nitrogen fixation apparatus (*nif* and *fix* genes) in bacteroids in response to low levels of free oxygen in infected plant cells (4).

Bacterial RNA polymerase (RNAP) sigma factor (σ) subunits control global transcription by determining promoter specificity (9, 10). The essential housekeeping sigma factor σ^70^ is encoded by *rpoD*. Alternative σs in Escherichia coli include RpoH (σ^32^), RpoS (σ^38/S^), RpoE (σ^24/E^), FecI (σ^Fec^), FliA (σ^28/F^), and, in some strains, RpoN (σ^54/N^). All E. coli σs except RpoN belong to the σ^70^ family, whose members contain up to four conserved structural domains (σ_1_ to σ_4_) (9); each directs RNAP core to a different promoter sequence (11). In E. coli, alternative σs generally respond to various physiological and environmental conditions: RpoH mediates response to heat shock, RpoS to nutrient limitation and other stresses, and FecI to iron limitation. RpoE (σ^24/E^), a member of the extracytoplasmic stress function (ECF) σ family, responds to cell envelope stresses such as periplasmic protein unfolding and outer membrane disruption (12). The anti-σ factor RseA sequesters RpoE at the cytoplasmic membrane in a transcriptionally inactive form. When the cell envelope perceives stress, RseA is degraded, freeing RpoE to associate with core RNA polymerase and change the transcriptional program, expressing genes from RpoE target promoters (13). In other bacteria, ECF σs like RpoE effect the appropriate transcriptional response to specific inputs—not all of them extracellular—which is why ECF σs are sometimes referred to as "group 4 σ factors" (14). ECF σs are the most abundant σ family; some bacterial genomes encode >100 ECF σs (15). At least 94 distinct groups have been defined within the ECF σ family, indicative of their broad diversity (16, 17).

In S. meliloti, genomic annotation discloses genes coding for the housekeeping σ, RpoD (σ^70^), RpoN, and two RpoH σs (18). Like other alphaproteobacteria, S. meliloti lacks an RpoS homolog (19). Instead, the RpoE2 ECF σ controls a large set of genes related to the general stress response (GSR) (20–22). While RpoN and RpoH1 are dispensable for growth in rich and defined media, they are required for effective symbiosis on Medicago host plants (23–26).

The S. meliloti genome also encodes 11 ECF-like σs (RpoE1 to -E10 and FecI). In this work, we systematically studied all 11 ECF-like σs. We used global transcription analyses to identify ECF σ target genes and putative ECF σ promoter motifs. Construction of mutants deleted for these ECF σs showed they have no major effects on free-living growth besides a slight decrease in growth rate. None of the ECF σs were required for symbiosis: a strain deleted for all ECF σ and anti-σ genes was symbiotically normal.

## RESULTS AND DISCUSSION

### Sinorhizobium meliloti strain Rm1021 possesses 11 ECF σ factors.

σ families are differentiated by the presence of four conserved structural domains (σ_1_ to σ_4_) (9). S. meliloti σ^70^ retains all four conserved domains. When we compared the proteins encoded by *rpoE1* to *rpoE10* and *fecI* with those of model sigma factors, we discovered that they, like other ECF σs, retain only domains σ_2_ and σ_4_ (9, 27). The genes encoding these ECF σs are dispersed among S. meliloti’s three replicons: six are chromosomal, four are on pSymB, and one is on pSymA (see Fig. S1A in the supplemental material).

10.1128/mSphereDirect.00454-18.1 FIG S1 Sinorhizobium meliloti sigma factors (σs) and anti-σs. (A) Genomic distribution of σ genes by replicon. Locations of ECF σ genes are indicated by blue triangles, RpoH σ genes by orange triangles, the RpoN σ gene by a red triangle, the RpoD housekeeping σ gene by a green triangle, and the RsiA2 orphan anti-σ gene by a yellow triangle. (B) Map of the *rpoE5* region on replicon pSymB showing two candidate anti-σ genes. Downstream of *rpoE5*, SMb21687 is annotated in the S. meliloti Rm1021 genome, while SMb23398, encoding an RsiA-like anti-σ protein, was additionally annotated in the S. meliloti Rm2011 genome (48). This *rsiA*-like ORF is located at positions 1405878 to 1406054 of pSymB. Transcription start sites (TSSs) identified by our 5′-RACE mapping and RNA-Seq (22, 48) are indicated by bent black arrows; TSSs identified only by RNA-Seq are indicated by bent dashed arrows (22), and a TSS identified only by 5′-RACE mapping is indicated by the bent gray arrow. (C) Amino acid sequence alignment of putative RsiA-like protein (SMb23398) with RsiA1 (SMc01505) and RsiA2 (SMc04884) of S. meliloti. Red shading indicates conserved in all 3 proteins, pink indicates conserved in 2, and blue indicates not conserved. Download FIG S1, JPG file, 0.8 MB. Copyright © 2018 Lang et al. 2018 Lang et al. This content is distributed under the terms of the Creative Commons Attribution 4.0 International license.

*rpoE10* was not initially annotated as a σ-encoding gene in S. meliloti (18, 28), but has been identified as such with the ECF*finder* webtool (http://ecf.g2l.bio.uni-goettingen.de:8080/ECFfinder/). Using ECF*finder*, we classified S. meliloti ECF σs into six of the 94 groups based on protein domain architecture, sequence similarity, genomic context, putative promoter motifs, and anti-σ features (16): ECF15 (RpoE2 and RpoE5), ECF16 (RpoE7), ECF26 (RpoE1, RpoE3, RpoE4, and RpoE6), ECF29 (RpoE8), ECF41 (RpoE9), and ECF42 (RpoE10). FecI is the unclassified exception.

**FIG 1:**
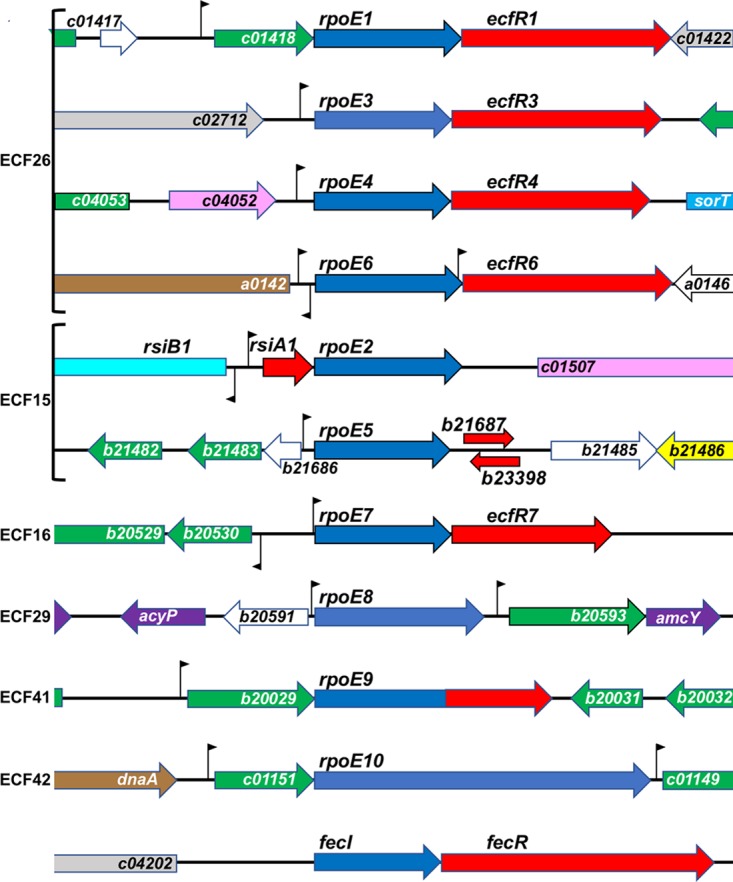
Genomic context of ECF σ genes in S. meliloti. σs are grouped according to the classification described in reference 16. Genomic context is anchored by blue ECF σs and red adjacent known and putative anti-σs. Abbreviated S. meliloti gene names indicate the replicon on which they are found: a, pSymA; b, pSymB; and c, chromosome. The likely *rpoE5* anti-σ lies downstream and in the same orientation as its partner σ; the overlapping arrow in the opposite orientation indicates an ORF with striking similarity to that of *rsiA1*, the *rpoE2* anti-σ (see Fig. S1 for more details). The anti-anti-σ (*rsiB1*) transcribed divergently from *rsiA1* is shown in turquoise. RpoE9 likely encodes its own anti-σ domain in the C-terminal half of its ORF. The flags dispersed throughout indicate the location and orientation of promoter motifs discussed in the text. The remaining colors follow the Riley classification convention found on the INRA Sinorhizobium meliloti 1021 website (https://iant.toulouse.inra.fr/bacteria/annotation/cgi/rhime.cgi), with several exceptions for clarity. The purple ORFs flanking *rpoE8* indicate small molecule metabolism, light gray indicates hypothetical partial homology, green indicates hypothetical global homology, white indicates unknown function, mauve indicates a not classified regulator, light blue indicates central intermediary metabolism, brown indicates macromolecule metabolism, and yellow indicates cell processes.

We examined the genomic context of each ECF σ (Fig. 1) to define its putative anti-σ and other neighboring genes. Anti-σs show diverse mechanisms for regulating ECF σ function; they typically display little if any sequence similarity (15, 27). Only about half of the 94 ECF σ groups are predicted to have a cognate anti-σ partner (17). We identified candidate anti-σs based on position within the same operon as, or closely linked to, the σ gene under examination; we considered the presence of a membrane-spanning domain to be incriminating, bearing in mind that not all anti-σs are membrane bound (see group ECF15 below).

We then systematically characterized transcriptomes of strains overexpressing individual σs (via a melibiose-inducible promoter [P*melA*] plasmid) compared to a control strain carrying the empty vector. We used S. meliloti CL150, a wild-type (WT) Rm1021 derivative corrected for nonfunctional *ecfR1* and *pstC* genes (22), which encode the RpoE1 anti-σ, and a subunit of the Pst high-affinity phosphate transporter, respectively, as our control strain. Rm1021-derived strains with an uncorrected *ecfR1* allele (CL150 and CL101) show high constitutive expression of RpoE1 target genes in agreement with our data from overexpressing RpoE1 via an exogenous promoter (see Data Set S1 in the supplemental material). Most expression changes attributed to the correction of *pstC* (i.e., those identified in CL150, but not CL101) are related to phosphate metabolism, as expected from previous studies (29, 30). We considered performing Affymetrix transcriptome analyses using a strain deleted for the corresponding σ/anti-σ pair as hosts for each overexpression plasmid but were concerned that unregulated ECF σ expression would be deleterious and wished to limit the number of control strains needed. Thus, one caution for interpretation of our Affymetrix transcriptomes is that our use of the WT host, which retains all the anti-σ genes, could preclude σ activation under the growth conditions used. Further limitations may apply; for example, other proteins besides anti-σs may negatively control interaction of σs with RNAP. Activation of some σs may require posttranslational modifications, such as phosphorylation (15). Finally, even if the active σ interacts with RNAP, subsequent target gene transcription may require an activator or inducer to relieve repression.

10.1128/mSphereDirect.00454-18.6 DATA SET S1 Affymetrix GeneChip analyses of strains overexpressing individual ECF σs. Download Data Set S1, XLSX file, 0.1 MB. Copyright © 2018 Lang et al. 2018 Lang et al. This content is distributed under the terms of the Creative Commons Attribution 4.0 International license.

**FIG 2:**
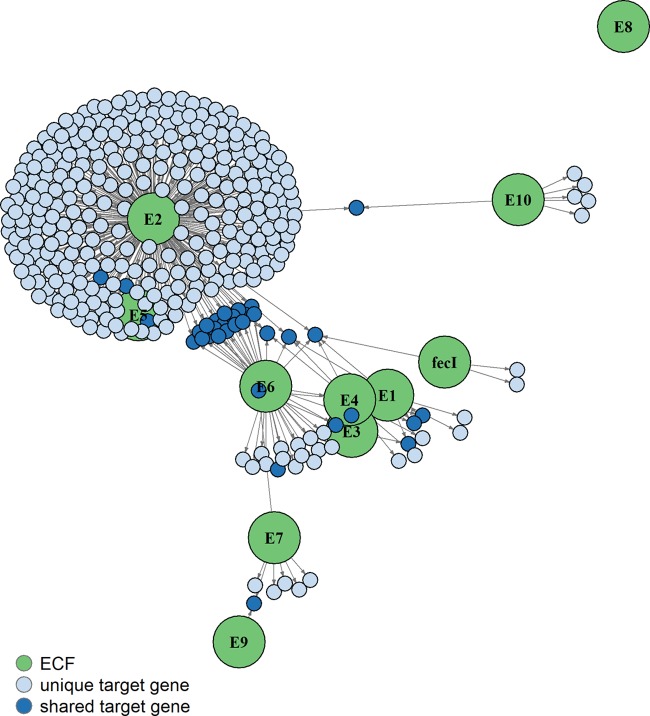
Network of ECF σs and their putative target genes. The network diagram, created with the R igraph package (90) and the Fruchterman-Reingold layout algorithm (91), is based on transcriptome data from Data Set S1. RpoE1 to RpoE10 (E1 to E10) and FecI are represented by green circles. Arrows of arbitrary length from each ECF σ point to putative target genes (blue circles) whose expression appears dependent on that particular σ. Light blue circles indicate target genes of only one σ, while dark blue circles indicate target genes of more than one σ. Since each ECF σ was overexpressed from an exogenous promoter, ECF σs are not included on the diagram as targets, even if demonstrated to autoregulate their own expression in other studies. Because the layout algorithm places features somewhat arbitrarily, some green circles such as those for RpoE5 and RpoE6 are partially obscured by blue circles representing their unique and shared target genes. The numbers of direct and indirect target genes for each σ as a result of this study are as follows: RpoE1, 3; RpoE2, 320; RpoE3, 4; RpoE5, 1; RpoE6, 40; RpoE7, 7; RpoE8, 0; RpoE9, 1; and RpoE10, 6.

**FIG 3:**
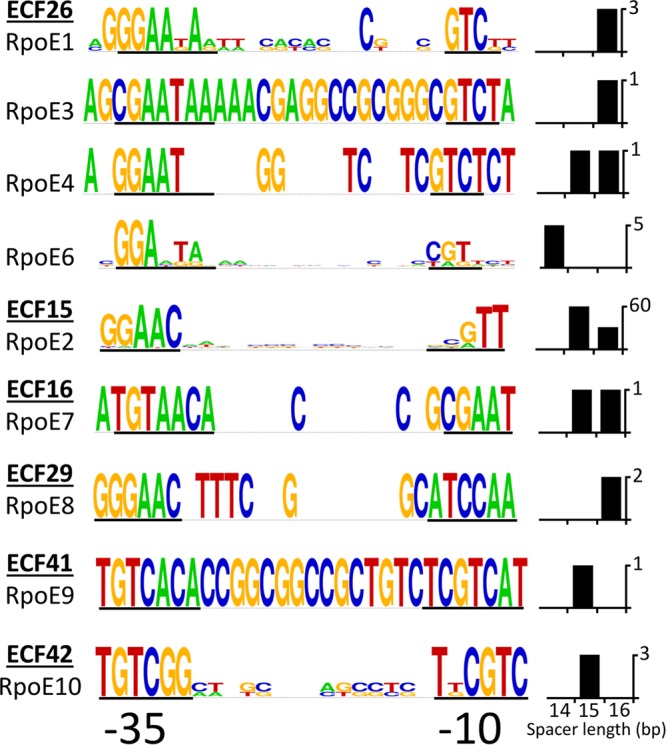
ECF σ −35 and −10 consensus promoter motifs. Motifs were identified from sequences upstream of transcription start sites (TSSs) of ECF σ-dependent target genes as described in Materials and Methods (Table 1). Sequence logos for predicted promoters were generated with WebLogo (https://weblogo.berkeley.edu). Promoters of genes that showed cross-regulation by multiple ECF σs were included in the sequence logo for only the ECF σ that showed the highest increase in expression of that target gene. The height of each letter in the sequence logo is proportional to the frequency of that nucleotide at that position, while the height of the entire stack is proportional to the sequence conservation at that position. Thus, logos generated from two sequences (RpoE4, RpE7, and RpoE8) will have blank spaces where no conservation is observed and letters of full height at the other positions. Similarly, logos generated from one sequence (RpoE3 and RpoE9) will have letters of full height at all positions. ECF σs are listed by their ECF group numbers; the number of upstream sequences used to develop each motif and their spacer lengths are indicated in the charts next to each logo. Sequences corresponding to the cross-species consensus motifs previously identified within the −35 and −10 regions are underlined in boldface (31). 5′-RACE mapping and RNA-Seq (22) of SMb20593, downstream of *rpoE8*, failed to identify its TSS; thus, a near perfect match to the putative RpoE8 promoter motif identified upstream of *rpoE8*, which is similar to consensus motifs identified for ECF29 family σs (31), was used to define the consensus.

Each of the ECF σ genes showed high expression from pCAP11 (Data Set S1), and the resulting transcriptomes let us identify candidate target genes for each ECF σ (Fig. 2; Data Set S1). With the exception of RpoE2 (the GSR σ), most S. meliloti ECF σs showed surprisingly small sets of target genes. We used 5′ rapid amplification of cDNA ends (5′-RACE) mapping (Table 1; see Data Set S2 in the supplemental material) and global transcription start site (TSS) data (22) to identify TSSs for target genes and predict consensus −35 and −10 promoter motifs (Fig. 3). One needs to keep in mind that TSSs identified under one growth condition may differ under other growth conditions, because a different σ factor may mediate transcription initiation. Our predicted promoter motifs were consistent with those previously predicted for the ECF σ groups found in S. meliloti (16, 17, 21, 31, 32).

**TABLE 1:** ECF σ-dependent promoters mapped in this study by 5′-RACE mapping or previously identified by RNA-Seq

Unique identifier^*a*^	Gene	Description	Log FC^*b*^	Sequence^*c*^
SMc01022		Cytochrome *b*-like protein	1.6, RpoE1; 1.9, RpoE3	GAAAGCGAATAAAAACGAGGCCGCGGGCGTCTAATCGG**A**
*SMc01021*		Conserved hypothetical protein	NC	
SMc01418		Hypothetical signal peptide protein	5.9, RpoE1; 2.7, RpoE4	GCCGGGAATAAATCCATAGCCCTCCGTGTCTTATCCTC**G**
*SMc01419*	*rpoE1*	RpoE1 σ factor	5.9, RpoE1	
SMc01420*	*ecfR1*	EcfR1 anti-σ factor	4.1, RpoE1	
SMc01421*	*ecfR1*	EcfR1 anti-σ factor	3.0, RpoE1	
SMc02156		Adhesin-like protein with periplasmic binding fold	3.6, RpoE1; 2.1, RpoE4	GAGGGAAGAATTGCGCCCTTCGAACAGTCGTTTCTCCT**G**
SMc04291		Dehydrogenase	0.8, RpoE1	GAAGGGAATAGTATGACACGGCGTTCCGTCTCACTGCG**A**
SMc02713	*rpoE3*	RpoE3 σ factor	3.7, RpoE3	CTTGCAGACTTAGGACCAAATGTTCCATATCATTGATG**G** (RpoD promoter motifs)
*SMc02714*^*d*^	*ecfR3*	EcfR3 anti-σ factor	NC	
SMb20556		Conserved hypothetical protein	1.0, RpoE2; 1.5, RpoE3; 1.4, RpoE4; 1.3, RpoE6; 1.5, RpoE7	CGTTGTTTTCTGGCCAGCGTGAGCATACCAGATCATGT**G** (RpoH2 promoter motifs)
SMc04049		Sulfite oxidase	0.8, RpoE1; 4.4, RpoE4; 2.7, RpoE6; 1.1, FecI	CGAGGGAATTTTCCGGGGCGTCAGTCGTCTCTTCCAGT**c**
*SMc04048*		Cytochrome *c*-like protein	4.9, RpoE4; 2.5, RpoE6	
*SMc04047*		Pseudoazurin	1.1, RpoE1; 4.5, RpoE4; 2.6, RpoE6; 0.9, FecI	
SMc04046		Conserved hypothetical protein	2.2, RpoE4	ACCTTCATGATTTACGTTGACCGACCTAAATCATGAAG**G** (RpoD/RpoH promoter motifs)
SMc04051	*rpoE4*	RpoE4 σ factor	1.8, RpoE1; 0.8, RpoE3; 6.1, RpoE4; 1.7, RpoE6	TCATGGAATAAGCGAGGCAGCTCGCTCGTCTCTACGCC**G**
*SMc04050*	*ecfR4*	EcfR4 anti-σ factor	1.7, RpoE1; 0.9, RpoE3; 4.1, RpoE4; 1.6, RpoE6	
SMb21484	*rpoE5*	RpoE5 σ factor	5.9, RpoE5; 4.8, RpoE2	CCTCAGGAACCAAAGGGCCGGAAAGGCATTTCCTAA**c**
*SMb21687*		EcfR5 anti-σ factor?	4.1, RpoE2	
SMa0143	*rpoE6*	RpoE6 σ factor	4.5, RpoE6	CATTGGACGATGAGACCGCTACCTGTAGATTGTGTCAG**a**
*SMa0144*	*ecfR6*	EcfR6 anti-σ factor	3.6, RpoE6	CTGCCGGAATAACACAGGCGACCGGACGTTCTCAGTCA***A***
SMa0139		Glyoxylase superfamily enzyme	2.6, RpoE6	GGATTGAATACTTTATGTACCCGTGCGACTTTCGAAAC**G**
SMa0142		Serine protease	0.7, RpoE4; 4.7, RpoE6	AAGAGGGAATAGACCGACGACTCAGCCGTTCTGACACA**a**
SMa_sRNA_10		sRNA	1.0, RpoE6	TTCGAAAGTCGCACGGGTACATAAAGTATTCAATCCGC**C**
SMa0148		Conserved hypothetical protein	6.5, RpoE6	ACGGAATAGAAGCCTCTCCGTTCCGTTACTCCCGGGCC***A***
SMa0187		Short-chain dehydrogenase	3.3, RpoE2; 2.5, RpoE6	TCGCCCAAACCTTTTGGCCTCGCCAACGTTCTACCTCC**t**
SMb20065		Hypothetical protein	3.3, RpoE2; 0.7, RpoE6	CAAAAGGAACTCCGGGCCCCCGGCCGCCGTTTCCGGGT**T**
SMb20074		Hypothetical protein	4.6, RpoE2; 0.9, RpoE6	GCCGATGGAACTTCGCCTACGGCTTCACGTTGCCCTCC**T**
*SMb20075*		Hypothetical protein	3.7, RpoE2	
SMb20522		PRC-barrel-domain protein	3.1, RpoE2; 1.9, RpoE6	TCGAAGGAACAAGTTGCCTGACGCCCCGTTAGGCACCT**g**
SMb20933	*exsG*	Sensor histidine kinase	3.6, RpoE2; 1.2, RpoE6	CGGACGGGGAACAAAGCAGCGGTCACTGCGTTTTTTGA**A**
*SMb20934*	*exsF*	Response regulator	1.7, RpoE2	
SMb21442		Hypothetical protein	4.4, RpoE2; 2.0, RpoE6	GGGGCGGAACAAATGGACGGTCGCGCCGTTTGAAACTC**G**
*SMb21441*		CBS-domain protein	3.8, RpoE2; 1.4, RpoE6	
SMc01509		Hypothetical protein	4.5, RpoE2; 2.9, RpoE6	TTACCGAAACAAATTCCTCCCTCATGCGTTGATCTAC**A*A***
*SMc01508*		Hypothetical protein	2.6, RpoE2; 1.4, RpoE6	
SMc01609	*ribH2*	6,7-Dimethyl-8-ribityllumazine synthase	0.6, RpoE6	AATTGTTCAGGGGCGTGAAATCCTTGGAAAATTCTGTC**G** (RpoD promoter motifs)
SMb20530		Conserved hypothetical membrane protein	6.8, RpoE7	AATGTAACATCGCTCCCGGTGGCTGCGAATGACGGACT***G***
*SMb20529*		Conserved hypothetical protein, DUF692 family	6.2, RpoE7	
*SMb20528*		Conserved hypothetical protein, DUF2063 family	5.1, RpoE7	
*SMb20527*		Conserved hypothetical protein	4.7, RpoE7	
SMb20531	*rpoE7*	RpoE7 σ factor	6.1, RpoE7	ACATGTAACAAGTAGCGAAACTCGGCGAATTGGGAGGA***A***
*SMb20532*	*ecfR7*	EcfR7 anti-σ factor	3.5, RpoE7	
SMb20592	*rpoE8*	RpoE8 σ factor	7.0, RpoE8	GGGAACATTTCCGGAGATAGGGCATCCAATATCCG***A***GA***A***
*SMb20593*^*e*^		Conserved hypothetical protein	NC	GGGAACGTTTCGAGCCGCGAAGCATCCAAAGCATGTCGT
*SMb20594*	*amcY*	Amicyanin	NC	
SMb20029		Carboxymuconolactone decarboxylase	0.8, RpoE7; 1.9, RpoE9	CCATGTCACACCGGCGGCCGCTGTCTCGTCATGGTGTC**G**
*SMb20030*	*rpoE9*	RpoE9 σ factor	5.0, RpoE9	
SMb20475		Conserved hypothetical protein	1.3, RpoE10	ACGATGTCGGATCGGTTGCGGCTGGTGCGTCATCGTATC**A**
*SMb20474*		Conserved hypothetical protein	1.1, RpoE10	
SMc01151		YCII-related protein	NC	TTTCGCCCCGCTTGTCGGCTATCAATAGCGCC***A***TTCGTC
*SMc01150*	*rpoE10*	RpoE10 σ factor	4.9, RpoE10	
SMc01149		Conserved hypothetical protein	1.6, RpoE10	CCCTGTCGGCAGGCGGCATCCTCCTTCGTCCTTGGAAT**g**
*SMc01148*		Conserved hypothetical protein	1.8, RpoE10	
SMc04203	*fecI*	FecI σ factor	5.7, FecI	No TSSs identified by RNA-Seq or 5′-RACE
*SMc04204*^*d*^	*fecR*	FecR anti-σ factor	NC	See Data Set S2^*e*^
*SMc04205*^*d*^		Iron/heme transport protein	NC	See Data Set S2^*e*^

a Previously reported RpoE2-dependent promoters are not shown, unless also identified as dependent on another ECF σ factor in this study. An identifier in italics indicates that the gene is predicted to be in an operon (22) with the gene(s) listed directly above. An asterisk indicates Affymetrix probe sets, designed for two putative pseudogenes of Rm1021, which hybridize to *ecfR1* mRNA in strains with a WT *ecfR1* allele.

b Log fold change of increased expression for ECF σ overexpression strains compared to the wild type. NC, no change. RpoE2 data were previously published (22); only those RpoE2-dependent genes whose promoter appears to be activated by other ECF σs in addition to RpoE2 are shown. Log FC is expressed as the log_2_ ratio of the change, i.e., a log FC of 1 equals a 2-fold change.

c Putative ECFσ-dependent promoters determined by 5′-RACE mapping, as described in Materials and Methods. The transcription start site (TSS) is in boldface. RNA-seq TSSs identified by Schlüter et al. (22) are in standard boldface, TSSs identified by 5′-RACE mapping are in italic boldface, and TSSs identified by both methods are in lowercase boldface. Sequences within putative −35 and −10 motifs, corresponding to the underlined cross-species consensus sequences in Fig. 3, are underlined. SMc04046, *rpoE3*, and *rpoE6* putative promoter regions have motifs similar to those found in promoters activated by RpoD and RpoH (22, 37, 38).

d Putative TSS was detected by 5′-RACE mapping, but no conserved promoter motifs were identified.

e 5′-RACE mapping and RNA-Seq (22) of SMb20593 failed to identify a TSS. We used sequence upstream of the SMb20593 ATG (start) codon (and 1 nt downstream of the *rpoE8* stop codon) for consensus motif development because it matches nearly perfectly with the putative RpoE8 promoter motif identified upstream of *rpoE8* and is similar to consensus motifs identified for group ECF29 σs (31).

10.1128/mSphereDirect.00454-18.7 DATA SET S2 ECF σ target genes with putative TSS but no predicted promoter motifs. Download Data Set S2, XLSX file, 0.0 MB. Copyright © 2018 Lang et al. 2018 Lang et al. This content is distributed under the terms of the Creative Commons Attribution 4.0 International license.

### Group ECF26 (RpoE1, RpoE3, RpoE4, and RpoE6).

All four members of group ECF26 are predicted to be coordinately expressed with their downstream anti-σ partners (Fig. 1) (33). The putative lipoprotein gene (SMc01418) upstream of *rpoE1* and *rpoE6*’s divergent expression from a serine protease gene (SMa0142) are arrangements widely conserved among other group ECF26 σs (16). In contrast, the genomic contexts of *rpoE3* and *rpoE4* do not appear to be conserved beyond the alphaproteobacteria (32). All four of their partner anti-σs (denoted EcfR1, EcfR3, EcfR4, and EcfR6) have an N-terminal anti-σ domain, a transmembrane domain, and a periplasmic C-terminal domain. This C-terminal domain has some similarity to that of Bacillus subtilis RsiW anti-σ, for which a promoter occlusion mechanism has been elucidated. However, such conservation is not proof of a conserved mechanism of activation (27); thus, the mechanism of S. meliloti ECF26 σ activation remains hypothetical.

RpoE1 and RpoE4 respond to sulfite compounds (thiosulfate and taurine) and activate expression of the *sorT-sorU-azu2* operon, whose proteins likely detoxify sulfite and contribute to sulfite respiration during stationary growth (32, 34–36). We found that RpoE1 activated expression of SMc02156, *rpoE4* to *ecfR4*, and its own operon (SMc01418-*rpoE1-ecfR1*), confirming the findings of Bastiat et al. (32), as well as two other genes (Fig. 2; Data Set S1). The *sorT-sorU-azu2* operon was the strongest RpoE4 target, as was found by Bastiat et al. Thus, RpoE1 and RpoE4 appear to cross-activate expression of their respective regulons (32) (Fig. 2; Data Set S1).

Overexpression of RpoE3 increased expression of four genes (Data Set S1), the strongest of which was SMc01022 (also a putative RpoE1 target); it encodes a YceJ family protein of unknown function with four transmembrane domains. RpoE3 does not appear to activate expression of its own operon, because RpoE3 overexpression failed to increase expression of the downstream anti-σ gene, *ecfR3*, and because its −35/−10 promoter motif is more similar to those recognized by RpoD and RpoH σs (Table 1) (22, 37, 38). We found that *rpoE3* expression was at least partially dependent on RpoH1 during heat shock (37), which is interesting as a possible connection between these regulatory circuits.

The RpoE6 overexpression transcriptome yielded the second largest set of ECF σ target genes after RpoE2; expression of 40 genes was increased ≥1.5-fold (Fig. 2; Data Set S1). RpoE6 target genes substantially overlapped with the RpoE2 regulon (*n* = 27; 67%), but most (21 of 27) showed lower expression when activated by RpoE6. The basis for this overlap is unknown since RpoE2 does not appear to activate *rpoE6* expression. Expression of the downstream anti-σ gene, *ecfR6*, increased only when we overexpressed RpoE6, arguing for autogenous regulation of the *rpoE6-ecfR6* operon; however, besides being cotranscribed with *rpoE6*, *ecfR6* may also be transcribed from an RpoE6-dependent promoter located within the *rpoE6* open reading frame (ORF) (Fig. 1 and Table 1). The most prominent RpoE6 targets lie near RpoE6 on pSymA: SMa0139 (glyoxylase superfamily enzyme), SMa0142 (serine protease), and SMa0146 and SMa0148 (hypothetical proteins) (Fig. 1). RpoE6 overexpression also activated expression of *rpoH2* and the *rpoE4-ecfR4* and *sorT-sorU-azu2* operons. Microarray data have so far failed to define a functional role for RpoE6, whose unique target genes were not activated by stresses that trigger expression of *rpoE2* and its regulon (21).

Our Affymetrix GeneChip also contains probe sets corresponding to tiled intergenic regions (IGRs) that are ≥150 bp (39). Previously, we showed that IGR expression data from the RpoE2 transcriptome could be correlated with transcriptome sequencing (RNA-Seq) data to identify noncoding, small RNAs (sRNAs) and previously unannotated open reading frames (ORFs) (22). Similarly, we correlated increased expression of the positive strand of the SMa0139-SMa0142 IGR to a previously identified sRNA (SMa_sRNA_10) encoded on the strand opposite the RpoE6 targets, SMa0139 and SMa0142 (Data Set S1) (22). The putative SMa_sRNA_10 promoter (Table 1) does not match those regulated by RpoE6, nor any other ECF σs; whether this sRNA is directly regulated by RpoE6 or regulates expression of ECF σ genes or their targets remains to be shown.

Generally, the −35/−10 promoter consensus motifs based on our sets of group ECF26 putative targets (Fig. 3) match the cross-species consensus (−35 GGAATA/−10 GT) determined earlier (17, 31). Consistent with the overlap mentioned above, the RpoE6 −35/−10 consensus motif shows some similarity to that of group ECF15, to which RpoE2 belongs.

### Group ECF15 (RpoE2 and RpoE5).

The GSR in alphaproteobacteria is mediated by some but not all group ECF15 σs (20, 40, 41). RpoE2 is the GSR σ factor in S. meliloti (21, 42). It is active and bound to RNAP during stationary-phase growth (32) and alters transcription in response to oxidative, osmotic, heat, desiccation, and starvation stresses, but is not required for symbiosis (43–46). Activity of alphaproteobacterial GSR σs is regulated via a partner-switching mechanism: a cytoplasmic anti-σ (RsiA1/RsiA2 in S. meliloti) sequesters σ from interacting with RNAP, until an anti-anti-σ (RsiB1/RsiB2) is activated by phosphorylation of its receiver domain (42, 47). Such phosphorylation allows anti-anti-σ to bind the anti-σ, releasing σ for interaction with RNAP (20, 47).

Consistent with its crucial role in the GSR, RpoE2 appears to directly activate expression of >100 genes (21, 22, 48, 49). We reanalyzed our previously reported RpoE2 Affymetrix transcriptome data set (22) using the analyses described in Materials and Methods, including a lower ≥1.5-fold cutoff, and identified 320 protein-coding genes whose expression increased, 93 of which have RpoE2-promoter motifs upstream of a TSS (Fig. 2 and 3; Data Set S1). RpoE2 sits at the top of a regulatory cascade: its direct targets include genes encoding σs (*rpoH2* and *rpoE5*) and two-component systems (*exsF*/*exsG* and SMa0113/SM0114); the latter pair is important for succinate-mediated catabolite repression (50). Like RpoE2, RpoH2 appears to play an important role in stationary-phase growth; its previously identified targets include many whose expression increases upon osmotic stress (37). Most RpoE2-dependent genes still lack a predicted function. Among transcripts whose expression changed with RpoE2 overexpression, a significant proportion (∼30%) showed a decrease compared to the control, albeit with most decreasing less than 2-fold: this may result from directing cellular physiology and metabolism toward that of the GSR (for example, via cascade regulation), or may simply be a response to inappropriate overexpression of RpoE2. In addition, RpoE2 was reported to activate expression of seven noncoding sRNAs (22), although the regulatory impact of these sRNAs is still unknown.

As for RpoE5, the other group ECF15 member in S. meliloti (21), its overexpression revealed only a single putative target: expression of SMb20091, encoding a conserved hypothetical protein, increased 1.7-fold (Fig. 2; Data Set S1). While RpoE2 also activates expression of SMb20091, we believe this is an indirect effect because SMb20091 lacks an upstream RpoE2-like promoter motif (Data Set S2). The paucity of RpoE5 targets in S. meliloti contrasts with that seen in Rhizobium etli; its two group ECF15 σs act in parallel, rather than in series, to regulate both unique and overlapping sets of genes (51).

Since RpoE2 activates *rpoE5* expression, we expected RpoE5 would be active under conditions where we know RpoE2 is active, but it is formally possible that RpoE5 was inactive under those conditions. Little is known about regulation of RpoE5 activity. We designated SMb21687, the gene downstream of *rpoE5* (Fig. 1), as its putative anti-σ factor because both were activated by RpoE2 (21). Closer inspection identified an unannotated ORF on the opposite strand of SMb21687, which encodes a protein similar to the RpoE2 anti-σs RsiA1/RsiA2 (Fig. S1B and S1C). While TSS mapping suggests that both of these ORFs are transcribed, further work is needed to dissect the significance of such findings and to determine if RpoE2 is involved in their regulation (Data Set S2).

### Group ECF16 (RpoE7).

RpoE7 appears to activate its own promoter because expression of downstream *ecfR7*, encoding its presumptive anti-σ, increases when RpoE7 is overexpressed (Data Set S1). The most highly expressed RpoE7 targets are four genes divergently transcribed from *rpoE7-ecfR7*, which encode conserved hypothetical proteins (SMb20527-SMb20530) Fig. 1 and 2; Data Set S1). Some S. meliloti strains such as Rm41 and the closely related species Sinorhizobium medicae lack *rpoE7-ecfR7* and SMb20527-SMb20530. However, orthologous proteins that are proposed to be involved in response to oxidative stress and heavy metals (chromate, dichromate, and cadmium) are present in the alphaproteobacterium Caulobacter crescentus (SigF-NrsF, CC3254-CC3257) (52, 53). We tested expression of *rpoE7* and its target, SMb20530, with promoter-*uidA* fusions, but failed to detect an increase in GUS expression after addition of H_2_O_2_ (1 mM), CdCl_2_ (50 and 100 µM), or K_2_CrO_4_ (50 and 100 µM) (data not shown). While the exact activation mechanism for group ECF16 σs is unknown, two cysteine residues in the C. crescentus NrsF anti-σ are required for its inactivation, leading to subsequent release of its partner SigF; these residues are conserved in S. meliloti RpoE7 (53). More distantly related group ECF16 σs in Bradyrhizobium diazoefficiens (EcfF and EcfS) have also been shown to be important for oxidative stress response and symbiosis (54, 55).

The two RpoE7 promoter motifs predicted upstream of *rpoE7-ecfR7* and SMb20527-SMb20530 differ substantially from those of the other S. meliloti ECFσs, but match well with group ECF16 σs from other organisms (Table 1 and Fig. 3) (17, 31).

### Group ECF29 (RpoE8).

To our knowledge, no group ECF29 σs have been studied in detail (17). S. meliloti *rpoE8* is located upstream of genes encoding a putative outer membrane protein (SMb20593) and a blue copper-like protein (AmcY) that may be involved in intermolecular electron transfer reactions (Fig. 1). These three genes are found close to each other in the genomes of many plant-nodulating bacteria, but are uncommon outside that group. *acyP*, encoding a putative acylphosphatase, is divergently transcribed from *rpoE8* in Sinorhizobium strains. Our *in silico* analyses suggest that SMb20591, annotated upstream of *acyP* and between *acyP* and *rpoE8*, is a pseudogene. No candidate anti-σs have been identified for group ECF29 σs, and SMb20593 lacks features of known anti-σs (17); thus, how RpoE8 activity is regulated remains a mystery.

Despite overexpressing *rpoE8* 126-fold compared to the control strain, we failed to detect a single RpoE8 target (Fig. 2; Data Set S1). Because no TSSs had been identified upstream of *rpoE8*, SMb20593, *amcY*, or *acyP*, we attempted to map TSSs upstream of these four genes. We identified two TSSs upstream of *rpoE8* and one upstream of *acyP*. One of the *rpoE8* TSSs had a −35/−10 motif identical to that identified by cross-species comparison of other group ECF29 σ genes (−35 GGGAAC/−10 GCATCCAA) (Table 1) (31), but we failed to identify any promoter motifs upstream of the other two TSSs (Data Set S2), despite the fact that by visual inspection we found a perfect RpoE8 −35/−10 match in the 77-nucleotide (nt) *rpoE8*-SMb20593 intergenic region (Table 1). Since overexpression of RpoE8 failed to increase expression of SMb20593, the significance of this motif in that location remains to be determined.

### Group ECF41 (RpoE9).

We identified a single RpoE9 target, SMb20029, a putative carboxymuconolactone decarboxylase with a conserved CxxC motif, suggestive of a responsive role to oxidative stress (56). While this target is located upstream of and likely cotranscribed with *rpoE9*, it is unlikely to function as an anti-σ (Fig. 1 and 2; Data Set S1). Rhodobacter sphaeroides carries an orthologous group ECF41 operon; like that in S. meliloti, it is the sole target of its ECF41 σ (56). The R. sphaeroides and Bacillus licheniformis group ECF41 σs are probably regulated by their long C-terminal domains rather than a separate anti-σ, and S. meliloti RpoE9 contains a similar domain (Fig. 1) (56). We previously identified a single TSS (22) upstream of SMb20029-*rpoE9* whose −35/−10 motif matched that predicted for group ECF41 σs (Table 1 and Fig. 3) (17, 31, 56).

### Group ECF42 (RpoE10).

These σs are larger than most other ECF σs due to an extended C-terminal domain that encodes tetratricopeptide repeats that could mediate protein-protein interactions. That and the lack of identifiable anti-σs near group ECF42 σ genes suggest that their activity is regulated by their C-terminal domain (17). Similar to group ECF42 σs in other organisms, *rpoE10* is located downstream of a gene encoding a protein of unknown function (SMc01151) (Fig. 1).

Analysis of our Affymetrix transcriptome identified six genes whose expression increased when we overexpressed RpoE10 (Fig. 2; Data Set S1). Four, predicted to be in two operons (SMc01149-SMc01148 and SMb20475-SMb20474), showed expression increases of 2-fold or greater. All four encode conserved hypothetical proteins: SMc01149 has a domain predicted to bind hydrophobic ligands, SMb20474 lacks a predicted function, and SMc01148 and SMb20475 are predicted to contain glyoxylase-like domains. Since SMc01151-*rpoE10* are predicted to be cotranscribed (22), but SMc01151 expression did not increase when we overexpressed RpoE10, RpoE10 apparently fails to activate its own expression by initiating transcription upstream of the first gene (SMc01151) of the putative operon. We previously identified a −35/−10 promoter motif upstream of the SMc01149 and SMb20475 TSSs (Fig. 1 and Table 1) (22) that matches motifs predicted for group ECF42 σs (17, 31). We used 5′-RACE mapping to identify a motif that overlaps the putative SMc01151 TSS and that is nearly identical to the SMc01149 upstream motif (Table 1). The fact that the predicted SMc01151 motif overlaps with its TSS could explain why SMc01151-*rpoE10* failed to show RpoE10-dependent overexpression.

The genomic context of S. meliloti RpoE10 is similar to that seen in Pseudomonas putida ECF10, the only other group ECF42 σ characterized to date; it deals with antibiotic stress resistance and biofilm formation (57).

### Unclassified (FecI).

ECF*finder* failed to assign FecI to any of the 94 ECF groups—even groups ECF05 to ECF10, which include FecI-like σs. S. meliloti FecI is closest to group ECF09, although its score is below that of true ECF09 σs (D. Pinto and T. Mascher, personal communication), which include Pseudomonas aeruginosa PvdS and Pseudomonas fluorescens PbrA, involved in iron uptake (17). With an FecR-like putative anti-σ encoded downstream of *fecI* (Fig. 1) (16, 17), the genomic context of *fecI* is more similar to those of groups ECF05 to ECF07 than to group ECF09. SMc04205, downstream of the *fecIR* operon, encodes a protein similar to TonB-dependent receptors of iron-containing proteins such as hemoglobin, leghemoglobin, transferrin, and lactoferrin. We identified only two putative FecI target genes: the RpoE4 target gene *sorT* and SMc04206, which encodes a putative extracellular protein with no predicted function. SMc04206 showed increased expression during iron limitation (58), consistent with a role for FecI in iron metabolism. Given its predicted role in iron uptake and its location downstream of *fecIR*, it was surprising that expression of SMc04205 showed no increase during iron limitation (58), or when we overexpressed FecI. Perhaps, as in E. coli, an additional extracellular signal is needed to trigger a FecR-mediated protein-protein interaction activation cascade (15).

We mapped putative TSS upstream of *fecR* and SMc04205, but could not identify any promoter motifs upstream of those genes (Table 1; Data Set S2). We also inspected DNA sequences upstream of the *fecI*, *fecR*, SMc04205, and SMc04206 translational starts for AT-rich −35/−10 promoter motifs similar to those of group ECF05 to -10 σs (17, 31, 59), but found no matching motifs.

### *S. meliloti* ECF σs are dispensable for nitrogen-fixing symbioses of *M. sativa* and *M. truncatula*.

To test if ECF σs play a role in symbiosis, we first created single and double insertions in ECF σ genes using nonreplicating plasmids (data not shown). Because (i) some of the insertions conferred polar effects on adjacent genes, (ii) when more than one plasmid insertion integrates into the genome, it allows their similar DNA sequences to promote genome rearrangements, and (iii) limited availability of antibiotic resistance markers precluded construction of a strain carrying more than a few ECF σ gene mutations, we switched to a precise deletion strategy. We constructed 12 strains: 11 each lacked a different ECF σ gene and its adjacent presumptive anti-σ gene, and the 12th lacked the orphan *rsiA2* anti-σ gene (Fig. 1 and Table 2; see Fig. S1 and Table S1 in the supplemental material). We also constructed 66 strains representing all double deletion combinations of the 12 single deletions listed in Table S1. As we learned more during the course of this work, we realized that some of the genes we suspected to encode anti-σs likely do not. To distinguish these, we retained the “SM_” locus identifier of genes unlikely to encode anti-σs, whereas those for putative anti-σs were designated *ecfRx*, *rsiAx*, and *fecR* (Fig. 1; Table S1).

**TABLE 2:** Strains and plasmids used in this study

Strain or plasmid	Description	Reference
*S. meliloti* strains		
Rm1021	WT SU47; Sm^r^	92
CL101	Rm1021 *ecfR1* corrected Sm^r^	22
CL150	Rm1021 *ecfR1 pstC* corrected Sm^r^	22
CL309	CL150 *nifD*::Tn*5-233* Sp^r^ Sm^r^	This study
RFF702	CL150 Δ*rpoE1-ecfR1* Sm^r^	This study
RFF164	CL150 Δ*rpoE2-rsiA1* Sm^r^	This study
RFF716	CL150 Δ*rpoE3-ecfR3* Sm^r^	This study
RFF165	CL150 Δ*rpoE4-ecfR4* Sm^r^	This study
RFF272	CL150 Δ*rpoE5*-SMb21687 Sm^r^	This study
RFF117	CL150 Δ*rpoE6-ecfR6* Sm^r^	This study
RFF344	CL150 Δ*rpoE7-ecfR7* Sm^r^	This study
RFF465	CL150 Δ*rpoE8*-SMb20593 Sm^r^	This study
RFF343	CL150 Δ*rpoE9*-SMb20029 Sm^r^	This study
RFF198	CL150 Δ*rpoE10-ecfR10* Sm^r^	This study
RFF300	CL150 Δ*fecI-fecR* Sm^r^	This study
RFF118	CL150 Δ*rsiA2* Sm^r^	This study
RFF625c	CL150 Δall-ECF σs/putative anti-σs Sm^r^	This study
RFF155	CL150 Δ*rpoH2*	This study
RFF157	CL150 Δ*rpoH1*	This study
RFF231	CL150 Δ*rpoH1* CL150 Δ*rpoH2*	This study
RFF299	CL150 Δ*rpoH1* CL150 Δ*rpoE2*	This study
Plasmids		
pCAP11	Broad-host-range expression vector, melibiose inducible; Sp^r^	76
pF1087	pCAP11 *rpoE1* Sp^r^	This study
pF1084	pCAP11 *rpoE2* Sp^r^	22
pF1071	pCAP11 *rpoE3* Sp^r^	This study
pF1085	pCAP11 *rpoE4* Sp^r^	This study
pF1074	pCAP11 *rpoE5* Sp^r^	This study
pF1088	pCAP11 *rpoE6* Sp^r^	This study
pF1080	pCAP11 *rpoE7* Sp^r^	This study
pF1086	pCAP11 *rpoE8* Sp^r^	This study
pF1077	pCAP11 *rpoE9* Sp^r^	This study
pCL139	pCAP11 *rpoE10* Sp^r^	This study
pF1082	pCAP11 *fecI* Sp^r^	This study
pCL308	pJQ200SK, to correct *mdh* mutation in RFF625; Gm^r^	This study
pF1323	pJQ200SK, to make Δ*rpoE1-ecfR1*; Gm^r^	This study
pF1332	pJQ200SK, to make Δ*rpoE2-ecfR2*; Gm^r^	This study
pF1322	pJQ200SK, to make Δ*rpoE3-ecfR3*; Gm^r^	This study
pF1328	pJQ200SK, to make Δ*rpoE4-ecfR4*; Gm^r^	This study
pF1340	pJQ200SK, to make Δ*rpoE5*-SMb21687; Gm^r^	This study
pF1324	pJQ200SK, to make Δ*rpoE6-ecfR6*; Gm^r^	This study
pF1343	pJQ200SK, to make Δ*rpoE7-ecfR7*; Gm^r^	This study
pF1351	pJQ200SK, to make Δ*rpoE8*-SMb20593; Gm^r^	This study
pF1342	pJQ200SK, to make Δ*rpoE9*-SMb20029; Gm^r^	This study
pF1333	pJQ200SK, to make Δ*rpoE10*-SMc01151; Gm^r^	This study
pF1341	pJQ200SK, to make Δ*fecI-fecR*; Gm^r^	This study
pF1326	pJQ200SK, to make Δ*rpoH1*; Gm^r^	This study
pF1327	pJQ200SK, to make Δ*rpoH2*; Gm^r^	This study
pJQ200SK	*sacB* vector; P15a *ori*; does not replicate in *S. meliloti*; Gm^r^	85
pRK600	ColE1; provides RK2 transfer functions; Cm^r^	93

10.1128/mSphereDirect.00454-18.4 TABLE S1 Deletions of individual ECF σ and putative anti-σ genes. Genes designated *ecfRx*, *fecR*, and *rsiAx* in the first column have *in silico* or experimental evidence suggesting that they encode anti-σ factors; those designated with SM identifiers were deleted based solely on their close proximity to a putative ECF σ gene. *rsiA2* encodes an orphan anti-σ gene not located near any σ gene. S. meliloti possesses three replicons: SMc, chromosome; SMa, pSymA megaplasmid; SMb, pSymB megaplasmid. The S. meliloti genome annotation is available at NCBI (accession no. NC_003047.1, NC_003037.1, and NC_003078.1) and https://iant.toulouse.inra.fr/bacteria/annotation/cgi/rhime.cgi. Download Table S1, DOCX file, 0.1 MB. Copyright © 2018 Lang et al. 2018 Lang et al. This content is distributed under the terms of the Creative Commons Attribution 4.0 International license.

We assayed all single and double deletions for nodulation and nitrogen fixation on two S. meliloti plant hosts: M. sativa (alfalfa) and M. truncatula (barrel medic). Nodulation was assessed by counting root nodules at 7 and 21 days postinoculation (dpi). Nitrogen fixation was assessed by nodule color and seedling appearance at 21 dpi. Nitrogen-fixing nodules are distinctly pink in color due to the presence of the oxygen-sequestering protein leghemoglobin (4), while nonfixing nodules are white or very pale pink. In addition, plants inoculated with nonfixing bacteria have yellowed and stunted shoots because they are nitrogen starved. All single and double ECF σ deletions elicited a normal symbiosis on both host plants (data not shown).

We also tested double mutants that had one of the Table S1 ECF σ deletions as well as either *rpoH1* or *rpoH2*. *rpoH1* mutants form nonfixing nodules, *rpoH2* mutants are like WT for symbiosis, and double *rpoH1 rpoH2* mutants form very few nodules (26; this study). All of our ECF σ *rpoH2* double mutants were symbiotically normal. All of our ECF σ *rpoH1* double mutants formed nonfixing nodules like their *rpoH1* parent, except for the *rpoE2 rpoH1* double mutant: it formed very few nodules, like the *rpoH1 rpoH2* mutant. This was expected, since RpoE2 is required to activate *rpoH2* expression (21).

Because none of the ECF σ/anti-σ pairs appeared essential for symbiosis when deleted singly or doubly, we constructed a strain deleted for all ECF σs and presumptive anti-σs (Table S1 [Materials and Methods]). Initial tests of this strain (RFF625) showed that it failed to fix nitrogen on host plants. Upon sequencing its genome, we determined its nonfixing phenotype was due to a point mutation in *mdh*, encoding malate dehydrogenase, an essential tricarboxylic acid (TCA) cycle gene. We corrected the *mdh* defect, creating RFF625c, and sequenced it and its CL150 WT parent. Our sequencing confirmed all expected deletions and corrections (*ecfR1*, *pstC*, and *mdh*), and also revealed two spontaneous nonsynonymous sequence variants not present in S. meliloti Rm1021 (see Table S2 in the supplemental material). Based on whole-genome sequencing of various S. meliloti lab strains, it is not unusual for new sequence variants to arise. It is formally possible that one or both of the RFF625c sequence variants could suppress its phenotypes, but nothing suggests that these genes (SMb20071 and SMb20811) are important for growth, stress response, or symbiosis. Correction of these variants would be required to confirm this assertion.

10.1128/mSphereDirect.00454-18.5 TABLE S2 Variants identified in S. meliloti CL150 and RFF625c, compared to Rm1021 resequencing data. All expected ECF σ/anti-σ deletions were confirmed in RFF625c. Note that our *ecfR1* correction was present in CL150, but not RFF625c, because *rpoE1-ecfR1* is deleted in RFF625c. SNV, single nucleotide variant. Ref., nucleotide in the Rm1021 reference sequence. Download Table S2, DOCX file, 0.1 MB. Copyright © 2018 Lang et al. 2018 Lang et al. This content is distributed under the terms of the Creative Commons Attribution 4.0 International license.

**FIG 4:**
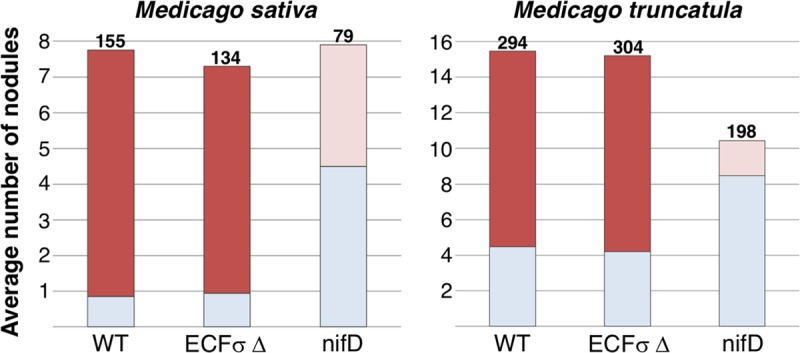
ECFσs are not required for symbiosis on M. sativa and M. truncatula. Nodulation assays were performed as described in Materials and Methods. The *y* axis indicates the average number of nodules per plant, 21 days after inoculation. The number of putative nitrogen-fixing nodules is indicated in red, and the number of small, white (nonfixing) nodules observed for each of the three bacterial strains is indicated in pale blue. Nodules formed by the nonfixing *nifD* mutant (CL309) were small and either white or very pale pink. The total number of nodules is shown above each column. The numbers of M. sativa plants assayed for each strain in this representative experiment are as follows: CL150, 20; RFF625c, 20; and CL309, 10. The numbers of *M. truncatula* plants assayed are as follows: CL150, 19; RFF625c, 20; and CL309, 19.

We assayed RFF625c, the all-ECF σ deletion strain, for symbiosis as for the single and double mutants. Surprisingly, RFF625c behaved like the WT: it formed nitrogen-fixing nodules on both host plants, with nodulation efficiency similar to that of the WT (Fig. 4). We also tested the ability of RFF625c to compete for nodule occupancy in Medicago truncatula, when coinoculated with its corresponding WT strain, and saw no obvious difference between strains that correlated with either presence or absence of the ECF σ genes (data not shown). It remains possible that in different environments, or facing other challenges, differences in competitiveness or fitness might be found.

Although RFF625c showed no obvious symbiotic defects, we used our Affymetrix GeneChip to explore changes in gene expression in 25-day-old M. truncatula nodules. We also analyzed gene expression in the *rpoE3*-SMc02714 and *rpoE8*-SMb20593 deletion mutants, because previous transcriptome analyses showed that expression of *rpoE3* and *rpoE8* was enhanced in nitrogen-fixing nodules (39, 60). While there were many nonoverlapping changes in gene expression, with a surprising lack of corresponding phenotype among the four strains (see Fig. S2 and Data Set S3 in the supplemental material), most were very small (1.1- to 1.5-fold), in contrast to an *rpoH1* mutant control strain that showed many changes (Data Set S3). Our analysis method judged expression changes as low as 1.1-fold to be statistically significant, but such low changes are unlikely to be biologically relevant. Genes whose expression changed ≥1.5-fold between the WT and RFF625c included some of the expected σ and anti-σ genes (since they are deleted in RFF625c), as well as a few RpoE2 targets. Expression of *amcY* downstream of *rpoE8*-SMb20593 increased 2.6- and 3.2-fold in RFF625c and the *rpoE8*-SMb20593 deletion strain, respectively. The mechanism of increased *amcY* expression is unknown, but could occur because RpoE8 and/or SMb20593 represses *amcY*, or because the distance between *amcY* and an upstream promoter was decreased by deletion of *rpoE8*-SMb20593.

10.1128/mSphereDirect.00454-18.2 FIG S2 Affymetrix GeneChip analysis of nodule bacteria for ECF σ mutants. Global gene expression for each of three ECF σ mutant strains was compared to WT CL150. The Venn diagram illustrates the degree of overlap and the number of changes in gene expression for mutant strains RFF625c (all-ECF σ Δ [yellow circle]), RFF716 (Δ*rpoE3-ecfR3* [blue circle]), and RFF465 (Δ*rpoE8-*SMb20593 [green circle]). We used a 1.1-fold cutoff for gene expression changes because <20 genes in the combined data sets showed expression increases of ≥1.5-fold (Data Set S3). Download FIG S2, JPG file, 0.7 MB. Copyright © 2018 Lang et al. 2018 Lang et al. This content is distributed under the terms of the Creative Commons Attribution 4.0 International license.

10.1128/mSphereDirect.00454-18.8 DATA SET S3 Affymetrix GeneChip expression changes for bacteria in nodules elicited by σ deletion strains versus wild type. Download Data Set S3, XLSX file, 0.5 MB. Copyright © 2018 Lang et al. 2018 Lang et al. This content is distributed under the terms of the Creative Commons Attribution 4.0 International license.

In summary, we observed no significant differences in symbiosis between WT and RFF625c. We conclude that the only alternative σs required for symbiosis under laboratory conditions are RpoN and RpoH1, with RpoE2 and RpoH2 being more critical when RpoH1 is absent.

### The *S. meliloti* strain deleted for all ECF σs behaves like the wild type for most phenotypes tested in culture.

We monitored growth of RFF625c and WT CL150 in complex LB and minimal M9 sucrose media. We streaked both strains for single colonies on LB with streptomycin (LB+Sm) and M9 sucrose+Sm and incubated them at 30 and 37°C. Our usual growth temperature for S. meliloti is 30°C; *rpoH1* heat shock σ mutants fail to grow on LB at 37°C, but can still grow on M9 sucrose at that elevated temperature. Both strains grew well at both temperatures on both media, although on LB medium, RFF625c took slightly longer to form colonies than WT CL150 (∼3.5 days versus ∼3 days for WT). In contrast, WT CL150 and RFF625c showed similar growth curves in both LB and M9 sucrose liquid media (see Fig. S3 in the supplemental material).

10.1128/mSphereDirect.00454-18.3 FIG S3 Growth curves for WT strain CL150 and the all-ECF σ deletion mutant RFF625c. Strains were grown in LB and M9 sucrose liquid media (Materials and Methods). Download FIG S3, JPG file, 0.2 MB. Copyright © 2018 Lang et al. 2018 Lang et al. This content is distributed under the terms of the Creative Commons Attribution 4.0 International license.

Because ECF σs often mediate response to external stresses, we compared how RFF625c copes, relative to WT, with agents or conditions that provoke various stresses (Table 3). We exposed cells to the detergents sodium deoxycholate (DOC) and sodium dodecyl sulfate (SDS) to test for envelope stress: the effects of both detergents on RFF625c were indistinguishable from those on WT.

**TABLE 3:** Phenotypic tests of RFF625c mutant, deleted for all ECF σ and putative anti-σ genes

Test^*a*^	Result
Growth on LB agar plates	Slightly slower than WT CL150
Growth on M9 sucrose agar plates	Indistinguishable from WT CL150
Heat stress (37°C) on LB and M9	Indistinguishable from WT CL150
Envelope stress (0.1% DOC)	Indistinguishable from WT CL150
Envelope stress (3% or 10% SDS)	Indistinguishable from WT CL150
Oxidative stress, exponential phase (1 mM H_2_O_2_)	Indistinguishable from WT CL150
Oxidative stress, stationary phase (100 mM H_2_O_2_)	Indistinguishable from WT CL150
Swim motility	Indistinguishable from WT CL150
EPS production	Indistinguishable from WT CL150

a Experimental details are described in Materials and Methods. DOC, sodium deoxycholate; SDS, sodium dodecyl sulfate.

To test for effects of oxidative stress, we treated LB-grown exponential-phase cells with H_2_O_2_ (1 mM for 30 min) as previously described (61). We saw biological variability among our four experimental replicates, but no significant difference in survival between WT (survival ranged from 9 to 24%) and RFF625c (8 to 17%). We similarly tested the effect of H_2_O_2_ on two biological replicates of LB-grown stationary-phase cells (100 mM for 10 min) and similarly saw little difference in survival (WT, 40 and 56%; RFF625c, 45 and 50%; and our Δ*rpoE2* strain RFF164, 40 and 44%). Our Δ*rpoE2* stationary-phase results are consistent with those of Flechard et al. (45), but differ with respect to their WT strains: our CL150 strain shows a dramatic decrease in viability after 10 min, while their Rm1021 strain shows no loss of viability after 10 and 15 min. We think their use of different strains, which carry mutations in *ecfR1* and *pstC*, and a different growth medium likely contribute to our differences in results.

We tested swim motility and production of the exopolysaccharide succinoglycan (EPS-I), which is critical for symbiosis. In both cases, RFF625c swam as well as the WT, and produced an indistinguishable amount of exopolysaccharide (Table 3).

**FIG 5:**
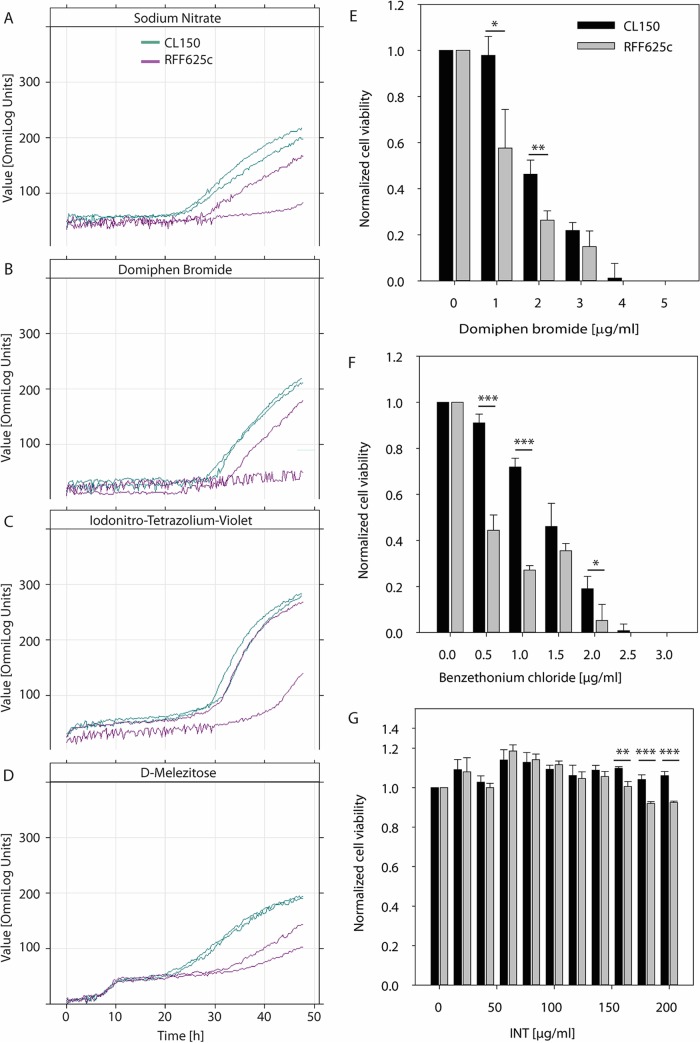
Comparison of the all-ECF σ deletion strain RFF625c to WT CL150 by Biolog Phenotype MicroArray and cell viability assays. Biolog kinetic plots, generated by Biolog OmniLog PM software, are shown in panels A to D for selected cultivation conditions. The conditions tested were growth in the presence of (A) 100 mM NaNO_3_ (Biolog plate PM09, well H06), (B) domiphen bromide (plate PM15, well D06), (C) iodonitrotetrazolium violet (INT; plate PM19, well D05), and (D) d-melezitose as the sole carbon source (plate PM02, well C04). Lines of the same color represent two biological replicates for CL150 (green) and RFF625c (purple). (E to G) Relative cell viabilities, determined as described in Materials and Methods, in the presence of domiphen bromide (E), benzethonium chloride (F), and INT (G). Cell viability measurements were normalized to the untreated CL150 control. Results showing statistically significant differences between the two strains, using a heteroscedastic, two-tailed *t* test, are indicated by asterisks (*, *P*  < 0.05; **, *P*  < 0.01; ***, *P*  < 0.001). Error bars indicate standard deviation from four replicates.

To test responses to diverse environmental conditions, we also used Biolog Phenotype MicroArrays (with PM software) (62) to compare RFF625c to the WT, assessing cellular respiration as a surrogate for growth under ∼1,900 test conditions (Materials and Methods). Such conditions included utilization of carbon, nitrogen, phosphorous, and sulfur sources, osmolytes, pH, and various chemical stresses. The standard analysis, using Biolog's proprietary Omnilog PM software, which relies on a subset of the available kinetic data, failed to identify any significant differences in cellular respiration between WT and RFF625c (Fig. 5A to D). Therefore, we further analyzed our Biolog data with the R opm package (63), which considers additional parameters, and can identify differences from kinetic curves that deviate from the sigmoid shape. The low number of replicates (*n =* 2) means the opm analysis was prone to errors but allowed discovery of potential phenotypes that would be validated upon further experimental testing. RFF625c showed 23 subtle differences compared to WT (Table 4; see Data Set S4 in the supplemental material). Under five conditions (addition of a fungicide, a disinfectant, a carbon source, a phosphorus source, and a sulfate source), the curves indicated that RFF625c had higher respiration than WT CL150. The diversity of these five conditions suggests they are false positives (Table 4). CL150 showed stronger respiration than RFF625c in the presence of 19 substances, including manganese, EDTA, quaternary ammonium compounds (domiphen bromide and benzethonium chloride); fluoroquinolones (ofloxacin, lomofloxacin, and enoxacin), several other antibiotics, and dyes (iodonitrotetrazolium violet and tetrazolium violet). Respiration was also slightly reduced in RFF625c with d-melezitose and with elevated levels of sodium nitrate or urea. Since multiple quaternary ammonium compounds and tetrazolium dyes had a stronger effect on the mutant than on WT, we retested RFF625c and the WT with different concentrations of these substances, assaying respiration afterwards using alamarBlue, a fluorescent dye. Domiphen bromide, benzethonium chloride, and iodonitrotetrazolium violet all had a stronger effect on RFF625c than on the WT in these viability assays, confirming the Biolog data (Fig. 5E to G).

**TABLE 4:** Biolog Phenotype MicroArray comparisons for WT strain CL150 compared to the all-ECF σ deletion strain RFF625c^*a*^

Plate, well^*b*^	Substrate^*c*^	AUC of CL150/AUC of RFF625c^*d*^	*P* value	Substrate description^*e*^
PM09, H06	Sodium nitrate (100 mM)	1.52	7.4E−08	Osmolyte
PM15, D06	Domiphen bromide no. 2	1.81	4.3E−07	Quaternary ammonium compound
PM19, D05	Iodonitrotetrazolium violet no. 1	1.44	7.4E−06	Tetrazolium dye
PM02, C04	d-Melezitose	1.56	1.2E−05	Carbon source, trisaccharide
PM20, H07	Tolylfluanid no. 3	0.43	3.7E−05	Antibacterial, phenylsulfamide
PM12, B12	Polymyxin B no. 4	1.70	8.9E−05	Antibacterial, cationic peptide-fatty acid
PM18, F08	Tinidazole no. 4	1.89	9.1E−05	Antibacterial, nitroimidazole
PM19, C04	Chlorhexidine no. 4	0.59	1.0E−04	Disinfectant, cationic bisbiguanide
PM12, E10	Benzethonium chloride no. 2	1.57	1.2E−04	Quaternary ammonium compound
PM04, E05	*O*-Phosphoryl-ethanolamine	0.58	3.3E−04	Phosphorous source
PM11, H12	Ofloxacin no. 4	1.48	4.1E−04	Antibacterial, fluoroquinolone
PM13, B04	Azlocillin no. 4	1.57	4.7E−04	Antibacterial, penicillin
PM11, D02	Capreomycin no. 2	1.69	1.3E−03	Antibacterial, cyclic peptide
PM11, E08	Enoxacin no. 4	1.95	2.1E−03	Antibacterial, fluoroquinolone
PM09, E08	Urea (3%)	1.45	5.2E−03	Osmolyte
PM13, G06	Manganese(II) chloride no. 2	1.30	5.5E−03	Heavy metal
PM11, B12	Lomefloxacin no. 4	1.40	6.2E−03	Antibacterial, fluoroquinolone
PM11, C03	Bleomycin no. 3	1.65	9.0E−03	Antibacterial, peptide- polyketide
PM19, A01	Josamycin no. 1	1.26	9.6E−03	Antibacterial, macrolide
PM04, A02	Sodium phosphate	1.35	1.0E−02	Phosphorous source
PM20, B09	Tetrazolium violet no. 1	1.40	2.4E−02	Tetrazolium dye
PM04, H11	Methane sulfonic acid	0.76	4.2E−02	Sulfur source
PM15, B06	EDTA no. 2	1.79	4.6E−02	Chelating agent

a Biolog Phenotype MicroArray comparisons were identified using the opm package (63 [see Materials and Methods]) for WT CL150 compared to the all-ECF σ deletion strain RFF625c.

b Shown are the plate number and then well number of Biolog Phenotype MicroArray 96-well plates. PM2, carbon sources; PM4, phosphorous and sulfur sources; PM9, osmolytes; PM11 to -20, chemical sensitivity tests for bacteria.

c For chemical stress tests, the number indicates which of the four concentrations (where 1 is lowest and 4 is highest) had a significant effect.

d AUC, area under the concentration-time curve (see Materials and Methods).

e Compounds listed as "antibacterial" possess antibacterial activity; however, their primary commercial use may not be treatment of bacterial infections (examples include tolylfluanid, tinidazole, and bleomycin).

10.1128/mSphereDirect.00454-18.9 DATA SET S4 Biolog Phenotype MicroArray data, analyzed by the opm R package (63), as described in Materials and Methods, for all compounds/wells on PM1 to PM20 plates. Download Data Set S4, XLSX file, 0.2 MB. Copyright © 2018 Lang et al. 2018 Lang et al. This content is distributed under the terms of the Creative Commons Attribution 4.0 International license.

Previously, Flechard et al. (44) found that the growth rate of an S. meliloti *rpoE2* mutant was reduced in comparison to WT Rm1021 at 0.5% NaCl, and Sauviac et al. (21) saw no loss of viability of an *rpoE2* mutant at up to 2.5 M (14.6%) NaCl. The Biolog system did not detect differences between WT CL150 and RFF625c at NaCl concentrations up to 10%. While the Biolog assay of RFF625c did not appear to have the same phenotype as the Rm1021-derived *rpoE2* mutant analyzed by Flechard et al., we did observe subtle respiratory defects with the osmolytes NaNO_3_ (100 mM) and urea (3%) (Table 4; Data Set S4), which may be due to lack of *rpoE2*. Overall, the differences between RFF625c and WT in Biolog assays were very subtle considering that the GSR RpoE2, which regulates >300 genes, is also deleted in RFF625c.

### Concluding remarks.

Rhizobia are known for the large size, complexity, and plasticity of their genomes (64); thus, it is unsurprising that many S. meliloti ECF σs would be retained to carry out narrow functions for adaptation to specific environmental conditions and that closely related Sinorhizobium species would differ in composition, regulation, and genomic contexts of ECF σs. In this study, we explored the S. meliloti ECF σ landscape using transcriptome analyses, TSS mapping, *in silico* analyses, and phenotypic tests of ECF σ mutants.

Our work shows that, except for RpoE2, which directly or indirectly alters expression of >300 genes (21, 22, 48), the ECF σ regulons comprise small numbers of genes when individual ECF σs are overexpressed in a WT background. While RpoE6 activates ∼40 genes, each of the remaining ECF σs increases expression of 10 or fewer genes. RpoE2, the GSR ECF σ, is thus, likely the only "core" ECF σ in S. meliloti, while the other ECF σs perform accessory roles that confer growth advantages in certain situations, but not for growth or symbiosis under laboratory conditions. A recent transposon sequencing (Tn-Seq) insertion study identified genes required for fitness during growth in rich and defined media (65): insertion in *fecI* was the only ECF σ gene insertion that conferred moderate growth impairment (and only in rich medium). This is consistent with our results, where a mutant deleted for all ECF σ genes was symbiotically normal and grew well under most conditions tested, and supports our conclusion that S. meliloti ECF σs are mostly dispensable.

Our study is the first report of an alphaproteobacterial strain deleted for all of its multiple ECF σs. The number of ECF σs encoded in alphaproteobacterial genomes varies widely: obligate intracellular species with reduced genomes such as Rickettsia, Wolbachia, and Liberibacter lack ECF σs (66); bartonellae have only a single group ECF15 σ, which is involved in the GSR and host adaptation (67, 68), and brucellae have two ECF σs—a group ECF16 σ and a group ECF15 σ involved in the GSR and mammalian infection (69). Model plant-associated rhizobia with expanded genomes, such as Sinorhizobium, Mesorhizobium, Rhizobium, and Bradyrhizobium possess up to 20 ECF σs (70). Group ECF15 GSR σs are the best-characterized ECF σs in rhizobia, but their apparent roles in symbiosis differ. GSR σs are not required for normal symbiosis in S. meliloti, R. etli, and Rhizobium leguminosarum bv. *viciae*, perhaps due to redundant regulatory systems (20). In contrast, B. diazoefficiens *ecfG* mutants show severe nodulation defects because a functional GSR is critical in early symbiosis (71), a group ECF16 σ (EcfS) is required for effective symbiosis in B. diazoefficiens (55), and ECF σs (EcfF and EcfQ/CarQ) also play a prominent role in the B. diazoefficiens oxidative stress response (54, 72).

Regulatory "cross talk" becomes a concern when multiples of the same family of regulators are encoded in a genome. ECF σ-promoter cross talk could result in coordinated activation of multiple regulons, while absence of cross talk sustains activation of single ECF σ regulons. A comprehensive exploration of cross talk between 43 ECF σ groups found that cross talk was limited (31). This is consistent with our transcriptome data: we saw limited cross talk, mainly between RpoE2 and RpoE6, and expression increases usually much greater for one overexpressed ECF σ promoter than for the cross talking σ.

This study rules out significant roles for S. meliloti ECF σs in surviving treatment with envelope-disrupting agents and the development of nitrogen-fixing root nodules, and it has created tools for continued research in these areas where much awaits discovery. Our ECF σ deletion strains may prove useful hosts for design of synthetic regulatory circuits. For example, a recent study reported the assembly of multiple ECF σs into regulatory cascades of various lengths, to create "autonomous timer circuits" (73). This report makes a substantial contribution to our understanding of S. meliloti regulatory circuits and its partition of transcriptional space.

## MATERIALS AND METHODS

### Strains and plasmids.

Table 2 shows key strains and plasmids used in this study. S. meliloti strains were grown in M9 sucrose (supplemented with 500 ng/ml biotin), LB (5 g/liter NaCl), or TY (tryptone-yeast extract) medium at 30°C, as described previously (74). E. coli strains were grown in LB medium at 37°C. Antibiotics were used at the following concentrations: ampicillin (Ap), 50 to 100 µg ml^−1^; chloramphenicol (Cm), 50 µg ml^−1^; gentamicin (Gm), 5 µg ml^−1^ for E. coli and 25 to 50 µg ml^−1^ for S. meliloti; hygromycin (Hy), 50 µg ml^−1^; kanamycin (Km), 25 to 50 µg ml^−1^ for E. coli; neomycin (Nm), 50 to 100 µg ml^−1^ for S. meliloti; spectinomycin (Sp), 50 µg ml^−1^ for E. coli and 50 to 100 µg ml^−1^ for S. meliloti; streptomycin (Sm), 500 µg ml^−1^ for S. meliloti; and tetracycline (Tc), 10 µg ml^−1^. Triparental conjugation transferred both replicative and nonreplicative plasmids to S. meliloti. Marked insertions and deletions were transferred between S. meliloti strains using N3 phage transduction (75). We used standard techniques for cloning and PCR amplification.

### Construction of plasmids bearing regulatable ECF σs.

A nested-PCR approach was used to clone each of the 11 S. meliloti ECF σs into pCAP11 (76) so that each is in the same context when overexpressed upon addition of melibiose. Early rounds of PCR used specific upstream primers that carried part of an optimized Shine-Dalgarno sequence, a 7-nt spacer sequence, the translation start codon, and 15 to 19 nt of the specific ORF being amplified (see Data Set S5 in the supplemental material). Two of the ECF σs (*rpoE6* and *rpoE7*) use GTG as their native start codon; this was changed to ATG for purposes of uniformity. Specific downstream primers contained an AvrII sequence and 17 to 20 nt of sequence complementary to and mostly downstream of the ORF termination codon.

10.1128/mSphereDirect.00454-18.10 DATA SET S5 Oligonucleotide primers used in this study. Sheet 1, PCR primers for plasmid and strain construction; Sheet 2, primers for 5′-RACE mapping. Download Data Set S5, XLSX file, 0.0 MB. Copyright © 2018 Lang et al. 2018 Lang et al. This content is distributed under the terms of the Creative Commons Attribution 4.0 International license.

Amplification of each σ gene was initiated using low levels (0.1 µM) of primers for 20 cycles; a universal pCAP11 primer (which added an AvrII site and completed the Shine-Dalgarno sequence) and the specific downstream primer were then added (to final concentrations of 0.45 and 0.55 µM, respectively), and amplification was continued for another 20 cycles. Purified PCR products were digested with AvrII and cloned into AvrII-digested pCAP11 to create a complete set of plasmids, each carrying a distinct ECF σ under the control of the melibiose-inducible promoter.

### Transcriptome analyses.

To identify genes whose expression was dependent on each of the 11 ECF σs, we used strains overexpressing ECF σs via the melibiose-inducible promoter (P*melA*) plasmids described above. Each plasmid was conjugated into the S. meliloti WT strain CL150, an Rm1021-derived strain corrected for mutations in *pstC* and *ecfR1* (Table 2; Data Set S5) (22). We employed three different control strains: CL150/pCAP11, CL101/pCAP11 (corrected only for *ecfR1*), and Rm1021/pCAP11.

For Affymetrix GeneChip experiments, we optimized growth conditions for expression of the ECF σs in S. meliloti. S. meliloti carrying ECF σ overexpression constructs grew well in M9 minimal medium with either 0.4% glycerol (to an optical density at 600 nm [OD_600_] of ∼6) or 0.4% succinate (to an OD_600_ of ∼1.7). We induced the *melA* promoter of the P*melA-rpoE2* strain with 0.4% melibiose when M9 glycerol-grown cells reached an OD_600_ of 0.5, and used real-time quantitative PCR (RT-qPCR), as previously described (39), to assay expression of *rpoE2* and two previously identified RpoE2 target genes, SMc00885 and SMb21456 (21). Expression of *rpoE2* and SMc00885 increased for 30 min after addition of melibiose, but no longer, while transcription of SMb21456 increased over the full 2-h time course.

RT-qPCR assays revealed that the *melA* promoter is leaky: *rpoE2* expression increased 37-fold under noninducing conditions compared to the pCAP11 control strain. We tested if growing cells in M9 succinate reduced background expression via catabolite repression, but background expression was not reduced, and melibiose induction was less efficient. We concluded that a 30-min melibiose induction of M9 glycerol-grown cells at an OD_600_ of 0.5 was appropriate for analysis of ECF σ-dependent gene expression.

Using these optimized conditions, we obtained six biological replicates of the CL150/pCAP11 control strain and three biological replicates for each of the remaining 13 strains, by growing 30-ml cultures in 250-ml baffled flasks at 30°C. We carried out cell harvest, RNA purification, cDNA synthesis, and hybridization of labeled cDNA to our custom Affymetrix Symbiosis Chip as described previously (39). We analyzed Affymetrix chips using the affy (77) and limma (78) R software packages. We normalized chips using the RMA algorithm (79). We considered probe sets to be differentially expressed if the adjusted *P* value (80) was below 0.05 and the log fold change was greater than 0.6 (1.5-fold change). We compared each of the 11 ECF σ-overexpression strains to CL150/pCAP11; we also compared CL150/pCAP11 to the singly corrected CL101/pCAP11 strain and to the parent strain, Rm1021/pCAP11 (Data Set S1). We previously reported our RpoE2 data set (22) and further mine the same Affymetrix CEL files using the analysis methods described above.

To compare the nodule transcriptomes of selected mutants, we grew Medicago truncatula (Gaertn.) cv. Jemalong on buffered nodulation medium agar plates and spot inoculated them essentially as described previously (81) 4 days after planting with WT CL150, the all-ECF σ deletion strain (RFF625c), the Δ*rpoE8*-SMb20593 mutant (RFF465), the Δ*rpoE3-ecfR3* mutant (RFF716), or the Δ*rpoH1* mutant (RFF157). We inoculated 22 plants for each of four replicates, and harvested nodules 25 days after inoculation. Nodule RNA purification, cDNA synthesis, cDNA amplification, and hybridization of labeled amplified RNA to our custom Affymetrix Symbiosis Chip were performed as described previously (82). To identify differentially expressed genes, we analyzed chips as described above, but with a 1.1-fold change cutoff and an adjusted *P* value cutoff of 0.05.

### Transcription start site and promoter consensus motif determination.

Candidate transcription start sites (TSSs) for ECF σs and their target genes were identified by performing 5′-RACE (5′ rapid amplification of cDNA ends) on a subset of ECF σ-dependent genes as described previously (37) and mining published TSS data (22). Gene-specific reverse transcription primers and primers for second round PCR amplification (PCR primers) are shown in Data Set S5.

Promoter consensus motifs for putative target genes of each ECF σ were generated by subjecting sequences upstream of the TSS to MEME (Multiple Em for Motif Elicitation) analyses (83), as described previously (37), and by comparison to cross-species promoter consensuses (16, 17, 31). Motifs shown in Fig. 3 were generated using WebLogo (https://weblogo.berkeley.edu) (84), and variable spacing between the −35 and −10 motifs was compensated for by manually adjusting spacer length as reported previously (31).

### Construction of ECF σ factor mutants.

We created unmarked precise deletions of each ECF σ gene and its known or putative anti-σ gene in CL150 using the *sacB* vector, pJQ200SK, and sucrose counterselection (85). We confirmed precise deletions by PCR. Primers used for plasmid construction and checking deletion strains are listed in Data Set S5.

To make double deletions, we simply mated a second deletion construct into a strain already bearing a deletion and repeated the process outlined above. Alternatively, we created N3 phage lysates (75) of single-crossover deletion strains and used them to transduce the single crossover into strains that already contained one or more ECF σ deletions. By successive rounds of deletion, we created a strain fully deleted for all 11 ECF σs: RFF625 (Table S1).

Genomic sequencing (see below) showed that RFF625 carried a point mutation in *mdh*, which encodes malate dehydrogenase and is required for effective symbiosis (86). To correct the *mdh* mutation, WT *mdh* was cloned into pJQ200SK to create pCL308, which was used to replace the mutated gene via *sacB* selection as described above. Gene replacement was verified by PCR amplification and sequencing of the PCR product: the corrected version was named RFF625c.

### Genome sequencing and variant detection.

We sequenced the complete genomes of Rm1021, CL150, and RFF625c via Illumina MiSeq technology at the Stanford Protein and Nucleic Acid Facility. Using Nextera kits (Illumina), we prepared sequencing libraries from genomic DNA purified with DNeasy blood and tissue kits (Qiagen). We used CLC Genomics Workbench software (Qiagen) to map paired-end sequence reads to the Rm1021 reference genome and identify single-nucleotide and structural variants. Since the published reference sequence contains errors, only some of which have been identified (48), we compared variants identified in CL150 and RFF625c to those identified in our resequenced strain, Rm1021. Variants identified in CL150 and RFF625c, but not the resequenced Rm1021 genome, are listed in Table S2.

### Nodulation assays.

We assayed all single and double deletion strains and the all-ECF σ deletion strain for nodulation and nitrogen fixation on two S. meliloti plant hosts, M. sativa (alfalfa) and M. truncatula [Gaertn.] cv. Jemalong (barrel medic), with CL150 as the WT control strain and CL309 (*nifD*::Tn*5-233*) as a non-nitrogen-fixing control strain. CL309 was made by transducing the Tn*5-233* from Rm1312-Sp (87) into CL150. We grew plants on nitrogen-free agar plates (10 to 20 plants per plate) as described above. We inoculated the root tips 2 days after planting with 1 µl washed cells diluted to an OD_600_ of 0.05. Nodulation was assessed by counting root nodules on each plant at 7 and 21 days postinoculation (dpi). Nitrogen fixation was assessed by nodule color and seedling appearance at 21 dpi. Nitrogen-fixing nodules are distinctly pink in color due to the presence of the oxygen-sequestering protein leghemoglobin (4), while nonfixing nodules are white or very pale pink. In addition, plants inoculated with nonfixing bacteria have yellowed and stunted shoots because they are nitrogen starved.

### Phenotypic comparisons between WT CL150 and the all-ECF σ deletion strain RFF625c.

We monitored growth of CL150 and RFF625c in LB and M9 sucrose liquid media at our usual growth temperature of 30°C as previously described (88). We also assayed growth and heat sensitivity on LB and M9 sucrose agar plates at 30 and 37°C. We determined H_2_O_2_ sensitivity of exponential and stationary-phase LB-grown cells as previously described (45, 61), treating the cells with 1 mM H_2_O_2_ for 30 min or with 100 mM H_2_O_2_ for 10 min, respectively. We tested sodium deoxycholate (DOC) sensitivity by spotting serial dilutions of log-phase MgSO_4_-washed cells onto LB+Sm agar plates containing 0.1% DOC and incubating them at 30°C, as previously described (89). We used a filter disc assay to test sodium dodecyl sulfate (SDS) sensitivity, spotting discs on lawns of each strain grown on LB with 2 µl of 3% and 10% SDS; after incubation at 30°C, we measured the resultant zones of inhibition. EPS-I was assayed on LB plates with 0.02% calcofluor white, and swim motility was assayed on soft agar plates, as previously described (89).

### Biolog Phenotype MicroArrays.

Biolog (Hayward, CA) ran Phenotype MicroArrays on our WT CL150 strain and all-ECF σ deletion (RFF625c) strains. Briefly, fresh colonies from LB plates were resuspended in proprietary Biolog media and dispensed into 96-well Biolog PM plates (PM1 to -20) to test ∼1,900 cultivation conditions, including sources of carbon, nitrogen, phosphorous, or sulfur, and challenge with osmolytes, pH, or chemical stresses. Strains were cultivated in duplicate using an OmniLog incubator. In addition to data generated by the Biolog OmniLog software, we received data as a .csv file, which we analyzed using the do_aggr (bootstrap = 100) and opm_mcp function of the opm package for R (63; https://www.dsmz.de/research/microorganisms/projects/analysis-of-omnilog-phenotype-microarray-data.html). *P* values were calculated using the R aov (analysis of variance) function. Because we assayed only two replicates of each strain, statistical analysis is error prone, yet provided a method to identify potential conditions under which growth levels of the WT and RFF625c strains are most likely to differ.

To validate some of the Biolog results, we inoculated four cultures of CL150 and RFF625c in M9 medium plus 0.4% glycerol to an OD_600_ of 0.1 from precultures in the same medium. After 20 h of cultivation at 30°C, the cultures were diluted to an OD of 0.1 with M9 medium. Ninety microliters of each cell suspension was mixed with 10 µl of aqueous dilutions of domiphen bromide, benzethonium chloride, and iodonitrotetrazolium violet in black microtiter plates. The mixtures were incubated for 60 min at 30°C, and 10 µl of a 1:1 (vol/vol) water-alamarBlue (Thermo Scientific) mixture was added. Fluorescence was measured at an excitation of 544 nm and emission of 590 nm immediately after addition of alamarBlue and after 60 min of incubation at 30°C. Viability was calculated by subtracting the zero time point fluorescent readings from the 60-min readings and normalized to the untreated WT control.

### Accession number(s).

The Affymetrix GeneChip data have been deposited in the Gene Expression Omnibus (GEO) database under Superseries accession no. GSE116680.

## References

[1] Jones KM, Kobayashi H, Davies BW, Taga ME, Walker GC 2007 How rhizobial symbionts invade plants: the *Sinorhizobium-Medicago* model. Nat Rev Microbiol 5:619–633. doi:10.1038/nrmicro1705. 17632573 PMC2766523

[2] Poole P, Ramachandran V, Terpolilli J 2018 Rhizobia: from saprophytes to endosymbionts. Nat Rev Micro 16:291–303. doi:10.1038/nrmicro.2017.171. 29379215

[3] Kondorosi E, Mergaert P, Kereszt A 2013 A paradigm for endosymbiotic life: cell differentiation of *Rhizobium* bacteria provoked by host plant factors. Annu Rev Microbiol 67:611–628. doi:10.1146/annurev-micro-092412-155630. 24024639

[4] Dixon R, Kahn D 2004 Genetic regulation of biological nitrogen fixation. Nat Rev Microbiol 2:621–631. doi:10.1038/nrmicro954. 15263897

[5] Long SR, Kahn ML, Seefeldt L, Tsay Y-F, Kopriva S 2015 Nitrogen and sulfur, p 711–768. *In* Buchanan BB, Gruissem W, Jones RL (ed), Biochemistry and molecular biology of plants. Wiley Blackwell, Oxford, United Kingdom.

[6] Barnett MJ, Fisher RF 2006 Global gene expression in the rhizobial-legume symbiosis. Symbiosis 42:1–24.

[7] Long SR 2016 SnapShot: signaling in symbiosis. Cell 167:582. doi:10.1016/j.cell.2016.09.046. 27716511

[8] Peck MC, Fisher RF, Long SR 2006 Diverse flavonoids stimulate NodD1 binding to *nod* gene promoters in *Sinorhizobium meliloti*. J Bacteriol 188:5417–5427. doi:10.1128/JB.00376-06. 16855231 PMC1540014

[9] Gruber TM, Gross CA 2003 Multiple sigma subunits and the partitioning of bacterial transcription space. Annu Rev Microbiol 57:441–466. doi:10.1146/annurev.micro.57.030502.090913. 14527287

[10] Österberg S, Del Peso-Santos T, Shingler V 2011 Regulation of alternative sigma factor use. Annu Rev Microbiol 65:37–55. doi:10.1146/annurev.micro.112408.134219. 21639785

[11] Feklistov A, Sharon BD, Darst SA, Gross CA 2014 Bacterial sigma factors: a historical, structural, and genomic perspective. Annu Rev Microbiol 68:357–376. doi:10.1146/annurev-micro-092412-155737. 25002089

[12] Lima S, Guo MS, Chaba R, Gross CA, Sauer RT 2013 Dual molecular signals mediate the bacterial response to outer-membrane stress. Science 340:837–841. doi:10.1126/science.1235358. 23687042 PMC3928677

[13] Ho TD, Ellermeier CD 2012 Extra cytoplasmic function σ factor activation. Curr Opin Microbiol 15:182–188. doi:10.1016/j.mib.2012.01.001. 22381678 PMC3320685

[14] Helmann JD 2002 The extracytoplasmic function (ECF) σ factors. Adv Microb Physiol 46:47–110. doi:10.1016/S0065-2911(02)46002-X. 12073657

[15] Mascher T 2013 Signaling diversity and evolution of extracytoplasmic function (ECF) σ factors. Curr Opin Microbiol 16:148–155. doi:10.1016/j.mib.2013.02.001. 23466210

[16] Staroń A, Sofia HJ, Dietrich S, Ulrich LE, Liesegang H, Mascher T 2009 The third pillar of bacterial signal transduction: classification of the extracytoplasmic function (ECF) σ factor protein family. Mol Microbiol 74:557–581. doi:10.1111/j.1365-2958.2009.06870.x. 19737356

[17] Pinto D, Mascher T 2016 The ECF classification: a phylogenetic reflection of the regulatory diversity in the extracytoplasmic function σ factor protein family, p 64–96. *In* de Bruijn FJ (ed), Stress and environmental regulation of gene expression and adaptation in bacteria, 1st ed. John Wiley & Sons, Inc, Hoboken, NJ.

[18] Galibert F, Finan TM, Long SR, Pühler A, Abola P, Ampe F, Barloy-Hubler F, Barnett MJ, Becker A, Boistard P, Bothe G, Boutry M, Bowser L, Buhrmester J, Cadieu E, Capela D, Chain P, Cowie A, Davis RW, Dréano S, Federspiel NA, Fisher RF, Gloux S, Godrie T, Goffeau A, Golding B, Gouzy J, Gurjal M, Hernandez-Lucas I, Hong A, Huizar L, Hyman RW, Jones T, Kahn D, Kahn ML, Kalman S, Keating DH, Kiss E, Komp C, Lelaure V, Masuy D, Palm C, Peck MC, Pohl TM, Portetelle D, Purnelle B, Ramsperger U, Surzycki R, Thébault P, Vandenbol M, Vorhölter FJ, Weidner S, Wells DH, Wong K, Yeh KC, Batut J 2001 The composite genome of the legume symbiont *Sinorhizobium meliloti*. Science 293:668–672. doi:10.1126/science.1060966. 11474104

[19] Battesti A, Majdalani N, Gottesman S 2011 The RpoS-mediated general stress response in *Escherichia coli*. Annu Rev Microbiol 65:189–213. doi:10.1146/annurev-micro-090110-102946. 21639793 PMC7356644

[20] Sauviac L, Bastiat B, Bruand C 2015 The general stress response in alpha-rhizobia, p 405–414. *In* de Bruijn FJ (ed), Biological nitrogen fixation, vol 1 John Wiley & Sons, Hoboken, NJ.

[21] Sauviac L, Philippe H, Phok K, Bruand C 2007 An extracytoplasmic function sigma factor acts as a general stress response regulator in *Sinorhizobium meliloti*. J Bacteriol 189:4204–4216. doi:10.1128/JB.00175-07. 17400745 PMC1913381

[22] Schlüter JP, Reinkensmeier J, Barnett MJ, Lang C, Krol E, Giegerich R, Long SR, Becker A 2013 Global mapping of transcription start sites and promoter motifs in the symbiotic alpha-proteobacterium *Sinorhizobium meliloti* 1021. BMC Genomics 14:156. doi:10.1186/1471-2164-14-156. 23497287 PMC3616915

[23] Mitsui H, Sato T, Sato Y, Ito N, Minamisawa K 2004 *Sinorhizobium meliloti* RpoH1 is required for effective nitrogen-fixing symbiosis with alfalfa. Mol Genet Genomics 271:416–425. doi:10.1007/s00438-004-0992-x. 15007732

[24] Oke V, Rushing BG, Fisher EJ, Moghadam-Tabrizi M, Long SR 2001 Identification of the heat-shock sigma factor RpoH and a second RpoH-like protein in *Sinorhizobium meliloti*. Microbiology 147:2399–2408. doi:10.1099/00221287-147-9-2399. 11535780

[25] Ronson CW, Nixon BT, Albright LM, Ausubel FM 1987 *Rhizobium meliloti ntrA* (*rpoN*) gene is required for diverse metabolic functions. J Bacteriol 169:2424–2431. doi:10.1128/jb.169.6.2424-2431.1987. 3034856 PMC212082

[26] Bittner AN, Oke V 2006 Multiple *groESL* operons are not key targets of RpoH1 and RpoH2 in *Sinorhizobium meliloti*. J Bacteriol 188:3507–3515. doi:10.1128/JB.188.10.3507-3515.2006. 16672605 PMC1482865

[27] Sineva E, Savkina M, Ades SE 2017 Themes and variations in gene regulation by extracytoplasmic function (ECF) sigma factors. Curr Opin Microbiol 36:128–137. doi:10.1016/j.mib.2017.05.004. 28575802 PMC5534382

[28] Becker A, Barnett MJ, Capela D, Dondrup M, Kamp PB, Krol E, Linke B, Rüberg S, Runte K, Schroeder BK, Weidner S, Yurgel SN, Batut J, Long SR, Pühler A, Goesmann A 2009 A portal for rhizobial genomes: RhizoGATE integrates a *Sinorhizobium meliloti* genome annotation update with postgenome data. J Biotechnol 140:45–50. doi:10.1016/j.jbiotec.2008.11.006. 19103235 PMC2656595

[29] Krol E, Becker A 2004 Global transcriptional analysis of the phosphate starvation response in *Sinorhizobium meliloti* strains 1021 and 2011. Mol Genet Genomics 272:1–17. doi:10.1007/s00438-004-1030-8. 15221452

[30] Yuan ZC, Zaheer R, Finan TM 2006 Regulation and properties of PstSCAB, a high-affinity, high-velocity phosphate transport system of *Sinorhizobium meliloti*. J Bacteriol 188:1089–1102. doi:10.1128/JB.188.3.1089-1102.2006. 16428413 PMC1347321

[31] Rhodius VA, Segall-Shapiro TH, Sharon BD, Ghodasara A, Orlova E, Tabakh H, Burkhardt DH, Clancy K, Peterson TC, Gross CA, Voigt CA 2013 Design of orthogonal genetic switches based on a crosstalk map of σs, anti-σs, and promoters. Mol Syst Biol 9:702. doi:10.1038/msb.2013.58. 24169405 PMC3817407

[32] Bastiat B, Sauviac L, Picheraux C, Rossignol M, Bruand C 2012 *Sinorhizobium meliloti* sigma factors RpoE1 and RpoE4 are activated in stationary phase in response to sulfite. PLoS One 7:e50768. doi:10.1371/journal.pone.0050768. 23226379 PMC3511301

[33] Krol E, Blom J, Winnebald J, Berhörster A, Barnett MJ, Goesmann A, Baumbach J, Becker A 2011 RhizoRegNet—a database of rhizobial transcription factors and regulatory networks. J Biotechnol 155:127–134. doi:10.1016/j.jbiotec.2010.11.004. 21087643

[34] Wilson JJ, Kappler U 2009 Sulfite oxidation in *Sinorhizobium meliloti*. Biochim Biophys Acta 1787:1516–1525. doi:10.1016/j.bbabio.2009.07.005. 19632192

[35] Low L, Ryan Kilmartin J, Paul VB, Ulrike K 2011 How are “atypical" sulfite dehydrogenases linked to cell metabolism? Interactions between the SorT sulfite dehydrogenase and small redox proteins. Front Microbiol 2:58. doi:10.3389/fmicb.2011.00058. 21833314 PMC3153034

[36] McGrath AP, Laming EL, Casas Garcia GP, Kvansakul M, Guss JM, Trewhella J, Calmes B, Bernhardt PV, Hanson GR, Kappler U, Maher MJ 2015 Structural basis of interprotein electron transfer in bacterial sulfite oxidation. eLife 4:e09066. doi:10.7554/eLife.09066. 26687009 PMC4760952

[37] Barnett MJ, Bittner AN, Toman CJ, Oke V, Long SR 2012 Dual RpoH sigma factors and transcriptional plasticity in a symbiotic bacterium. J Bacteriol 194:4983–4994. doi:10.1128/JB.00449-12. 22773790 PMC3430346

[38] MacLellan SR, MacLean AM, Finan TM 2006 Promoter prediction in the rhizobia. Microbiology 152:1751–1763. doi:10.1099/mic.0.28743-0. 16735738

[39] Barnett MJ, Toman CJ, Fisher RF, Long SR 2004 A dual-genome Symbiosis Chip for coordinate study of signal exchange and development in a prokaryote-host interaction. Proc Natl Acad Sci U S A 101:16636–16641. doi:10.1073/pnas.0407269101. 15542588 PMC527922

[40] Fiebig A, Herrou J, Willett J, Crosson S 2015 General stress signaling in the Alphaproteobacteria. Annu Rev Genet 49:603–625. doi:10.1146/annurev-genet-112414-054813. 26442844 PMC4710059

[41] Francez-Charlot A, Kaczmarczyk A, Fischer HM, Vorholt JA 2015 The general stress response in Alphaproteobacteria. Trends Microbiol 23:164–171. doi:10.1016/j.tim.2014.12.006. 25582885

[42] Bastiat B, Sauviac L, Bruand C 2010 Dual control of *Sinorhizobium meliloti* RpoE2 sigma factor activity by two PhyR-type two-component response regulators. J Bacteriol 192:2255–2265. doi:10.1128/JB.01666-09. 20154128 PMC2849433

[43] Barra-Bily L, Fontenelle C, Jan G, Flechard M, Trautwetter A, Pandey SP, Walker GC, Blanco C 2010 Proteomic alterations explain phenotypic changes in *Sinorhizobium meliloti* lacking the RNA chaperone Hfq. J Bacteriol 192:1719–1729. doi:10.1128/JB.01429-09. 20081032 PMC2832530

[44] Flechard M, Fontenelle C, Blanco C, Goude R, Ermel G, Trautwetter A 2010 RpoE2 of *Sinorhizobium meliloti* is necessary for trehalose synthesis and growth in hyperosmotic media. Microbiology 156:1708–1718. doi:10.1099/mic.0.034850-0. 20223801

[45] Flechard M, Fontenelle C, Trautwetter A, Ermel G, Blanco C 2009 *Sinorhizobium meliloti rpoE2* is necessary for H_2_O_2_ stress resistance during the stationary growth phase. FEMS Microbiol Lett 290:25–31. doi:10.1111/j.1574-6968.2008.01401.x. 19025578

[46] Humann JL, Ziemkiewicz HT, Yurgel SN, Kahn ML 2009 Regulatory and DNA repair genes contribute to the desiccation resistance of *Sinorhizobium meliloti* Rm1021. Appl Environ Microbiol 75:446–453. doi:10.1128/AEM.02207-08. 19028909 PMC2620701

[47] Sauviac L, Bruand C 2014 A putative bifunctional histidine kinase/phosphatase of the HWE family exerts positive and negative control on the *Sinorhizobium meliloti* general stress response. J Bacteriol 196:2526–2535. doi:10.1128/JB.01623-14. 24794560 PMC4097584

[48] Sallet E, Roux B, Sauviac L, Jardinaud MF, Carrère S, Faraut T, de Carvalho-Niebel F, Gouzy J, Gamas P, Capela D, Bruand C, Schiex T 2013 Next-generation annotation of prokaryotic genomes with EuGene-P: application to *Sinorhizobium meliloti* 2011. DNA Res 20:339. doi:10.1093/dnares/dst014. 23599422 PMC3738161

[49] Ichida H, Long SR 2016 LDSS-P: an advanced algorithm to extract functional short motifs associated with coordinated gene expression. Nucleic Acids Res 44:5045–5053. doi:10.1093/nar/gkw435. 27190233 PMC4914127

[50] Garcia PP, Bringhurst RM, Arango Pinedo C, Gage DJ 2010 Characterization of a two-component regulatory system that regulates succinate-mediated catabolite repression in *Sinorhizobium meliloti*. J Bacteriol 192:5725–5735. doi:10.1128/JB.00629-10. 20817764 PMC2953702

[51] Jans A, Vercruysse M, Gao S, Engelen K, Lambrichts I, Fauvart M, Michiels J 2013 Canonical and non-canonical EcfG sigma factors control the general stress response in *Rhizobium etli*. Microbiologyopen 2:976–987. doi:10.1002/mbo3.137. 24311555 PMC3892343

[52] Alvarez-Martinez CE, Baldini RL, Gomes SL 2006 A *Caulobacter crescentus* extracytoplasmic function sigma factor mediating the response to oxidative stress in stationary phase. J Bacteriol 188:1835–1846. doi:10.1128/JB.188.5.1835-1846.2006. 16484194 PMC1426549

[53] Kohler C, Lourenco RF, Avelar GM, Gomes SL 2012 Extracytoplasmic function (ECF) sigma factor σF is involved in *Caulobacter crescentus* response to heavy metal stress. BMC Microbiol 12:210. doi:10.1186/1471-2180-12-210. 22985357 PMC3511200

[54] Masloboeva N, Reutimann L, Stiefel P, Follador R, Leimer N, Hennecke H, Mesa S, Fischer HM 2012 Reactive oxygen species-inducible ECF σ factors of *Bradyrhizobium japonicum*. PLoS One 7:e43421. doi:10.1371/journal.pone.0043421. 22916258 PMC3420878

[55] Stockwell SB, Reutimann L, Guerinot ML 2012 A role for *Bradyrhizobium japonicum* ECF16 sigma factor EcfS in the formation of a functional symbiosis with soybean. Mol Plant Microbe Interact 25:119–128. doi:10.1094/MPMI-07-11-0188. 21879796

[56] Wecke T, Halang P, Staroń A, Dufour YS, Donohue TJ, Mascher T 2012 Extracytoplasmic function σ factors of the widely distributed group ECF41 contain a fused regulatory domain. Microbiologyopen 1:194–213. doi:10.1002/mbo3.22. 22950025 PMC3426412

[57] Tettmann B, Dotsch A, Armant O, Fjell CD, Overhage J 2014 Knockout of extracytoplasmic function sigma factor ECF-10 affects stress resistance and biofilm formation in *Pseudomonas putida* KT2440. Appl Environ Microbiol 80:4911–4919. doi:10.1128/AEM.01291-14. 24907323 PMC4135749

[58] Chao TC, Buhrmester J, Hansmeier N, Pühler A, Weidner S 2005 Role of the regulatory gene *rirA* in the transcriptional response of *Sinorhizobium meliloti* to iron limitation. Appl Environ Microbiol 71:5969–5982. doi:10.1128/AEM.71.10.5969-5982.2005. 16204511 PMC1265945

[59] Swingle B, Thete D, Moll M, Myers CR, Schneider DJ, Cartinhour S 2008 Characterization of the PvdS-regulated promoter motif in *Pseudomonas syringae* pv. tomato DC3000 reveals regulon members and insights regarding PvdS function in other pseudomonads. Mol Microbiol 68:871–889. doi:10.1111/j.1365-2958.2008.06209.x. 18363796

[60] Capela D, Filipe C, Bobik C, Batut J, Bruand C 2006 *Sinorhizobium meliloti* differentiation during symbiosis with alfalfa: a transcriptomic dissection. Mol Plant Microbe Interact 19:363–372. doi:10.1094/MPMI-19-0363. 16610739

[61] Lehman AP, Long SR 2013 Exopolysaccharides from *Sinorhizobium meliloti* can protect against H_2_O_2_-dependent damage. J Bacteriol 195:5362–5369. doi:10.1128/JB.00681-13. 24078609 PMC3837946

[62] Bochner BR 2009 Global phenotypic characterization of bacteria. FEMS Microbiol Rev 33:191–205. doi:10.1111/j.1574-6976.2008.00149.x. 19054113 PMC2704929

[63] Vaas LA, Sikorski J, Hofner B, Fiebig A, Buddruhs N, Klenk HP, Göker M 2013 opm: an R package for analysing OmniLog phenotype microarray data. Bioinformatics 29:1823–1824. doi:10.1093/bioinformatics/btt291. 23740744

[64] Batut J, Andersson SG, O'Callaghan D 2004 The evolution of chronic infection strategies in the alpha-proteobacteria. Nat Rev Microbiol 2:933–945. doi:10.1038/nrmicro1044. 15550939

[65] diCenzo GC, Benedict AB, Fondi M, Walker GC, Finan TM, Mengoni A, Griffitts JS 2018 Robustness encoded across essential and accessory replicons of the ecologically versatile bacterium *Sinorhizobium meliloti*. PLoS Genet 14:e1007357. doi:10.1371/journal.pgen.1007357. 29672509 PMC5929573

[66] Sällström B, Andersson SG 2005 Genome reduction in the alpha-proteobacteria. Curr Opin Microbiol 8:579–585. doi:10.1016/j.mib.2005.08.002. 16099701

[67] Abromaitis S, Koehler JE 2013 The *Bartonella quintana* extracytoplasmic function sigma factor RpoE has a role in bacterial adaptation to the arthropod vector environment. J Bacteriol 195:2662–2674. doi:10.1128/JB.01972-12. 23564167 PMC3676067

[68] Tu N, Lima A, Bandeali Z, Anderson B 2016 Characterization of the general stress response in *Bartonella henselae*. Microb Pathog 92:1–10. doi:10.1016/j.micpath.2015.12.010. 26724735 PMC4769946

[69] Kim HS, Caswell CC, Foreman R, Roop RM II, Crosson S 2013 The *Brucella abortus* general stress response system regulates chronic mammalian infection and is controlled by phosphorylation and proteolysis. J Biol Chem 288:13906–13916. doi:10.1074/jbc.M113.459305. 23546883 PMC3650426

[70] Masloboeva N, Hennecke H, Fischer HM 2015 Rhizobial extracytoplasmic function (ECF) σ factors and their role in oxidative stress response of *Bradyrhizobium japonicum*, p. 307–314. *In* de Bruijn FJ (ed), Biological nitrogen fixation, vol 1 John Wiley & Sons, Hoboken, NJ.

[71] Ledermann R, Bartsch I, Müller B, Wulser J, Fischer HM 2018 A functional general stress response of *Bradyrhizobium diazoefficiens* is required for early stages of host plant infection. Mol Plant Microbe Interact 31:537–547. doi:10.1094/MPMI-11-17-0284-R. 29278144

[72] Thaweethawakorn A, Parks D, So JS, Chang WS 2015 Role of the extracytoplasmic function sigma factor CarQ in oxidative response of *Bradyrhizobium japonicum*. J Microbiol 53:526–534. doi:10.1007/s12275-015-5308-9. 26224455

[73] Pinto D, Vecchione S, Wu H, Mauri M, Mascher T, Fritz G 2018 Engineering orthogonal synthetic timer circuits based on extracytoplasmic function sigma factors. Nucleic Acids 46:7450–7464. doi:10.1093/nar/gky614. PMC6101570 29986061

[74] Barnett MJ, Long SR 2015 The *Sinorhizobium meliloti* SyrM regulon: effects on global gene expression are mediated by *syrA* and *nodD3*. J Bacteriol 197:1792–1806. doi:10.1128/JB.02626-14. 25777671 PMC4402393

[75] Martin MO, Long SR 1984 Generalized transduction in *Rhizobium meliloti*. J Bacteriol 159:125–129. 6330025 10.1128/jb.159.1.125-129.1984 PMC215602

[76] Pinedo CA, Bringhurst RM, Gage DJ 2008 *Sinorhizobium meliloti* mutants lacking phosphotransferase system enzyme HPr or EIIA are altered in diverse processes, including carbon metabolism, cobalt requirements, and succinoglycan production. J Bacteriol 190:2947–2956. doi:10.1128/JB.01917-07. 18281401 PMC2293241

[77] Gautier L, Cope L, Bolstad BM, Irizarry RA 2004 affy—analysis of Affymetrix GeneChip data at the probe level. Bioinformatics 20:307–315. doi:10.1093/bioinformatics/btg405. 14960456

[78] Smyth GK 2005 Limma: linear models for microarray data, p. 397–420. *In* Gentleman R, Carey V, Huber W, Irizarry RA, Dudoit S (ed), Bioinformatics and computational biology solutions using R and Bioconductor. Springer-Verlag, New York, NY.

[79] Irizarry RA, Hobbs B, Collin F, Beazer-Barclay YD, Antonellis KJ, Scherf U, Speed TP 2003 Exploration, normalization, and summaries of high density oligonucleotide array probe level data. Biostatistics 4:249–264. doi:10.1093/biostatistics/4.2.249. 12925520

[80] Benjamini Y, Hochberg Y 1995 Controlling the false discovery rate: a practical and powerful approach to multiple testing. J R Statist Soc B 57:289–300.

[81] Mitra RM, Long SR 2004 Plant and bacterial symbiotic mutants define three transcriptionally distinct stages in the development of the *Medicago truncatula*/*Sinorhizobium meliloti* symbiosis. Plant Physiol 134:595–604. doi:10.1104/pp.103.031518. 14739349 PMC344536

[82] Lang C, Long SR 2015 Transcriptomic analysis of *Sinorhizobium meliloti* and *Medicago truncatula* symbiosis using nitrogen fixation-deficient nodules. Mol Plant Microbe Interact 28:856–868. doi:10.1094/MPMI-12-14-0407-R. 25844838

[83] Bailey TL, Williams N, Misleh C, Li WW 2006 MEME: discovering and analyzing DNA and protein sequence motifs. Nucleic Acids Res 34:W369–W373. doi:10.1093/nar/gkl198. 16845028 PMC1538909

[84] Crooks GE, Hon G, Chandonia JM, Brenner SE 2004 WebLogo: a sequence logo generator. Genome Res 14:1188–1190. doi:10.1101/gr.849004. 15173120 PMC419797

[85] Quandt J, Hynes MF 1993 Versatile suicide vectors which allow direct selection for gene replacement in Gram-negative bacteria. Gene 127:15–21. doi:10.1016/0378-1119(93)90611-6. 8486283

[86] Dymov SI, Meek DJ, Steven B, Driscoll BT 2004 Insertion of transposon Tn*5tac*1 in the *Sinorhizobium meliloti* malate dehydrogenase (*mdh*) gene results in conditional polar effects on downstream TCA cycle genes. Mol Plant Microbe Interact 17:1318–1327. doi:10.1094/MPMI.2004.17.12.1318. 15597737

[87] Barnett MJ, Swanson JA, Long SR 1998 Multiple genetic controls on *Rhizobium meliloti syrA*, a regulator of exopolysaccharide abundance. Genetics 148:19–32. 9475718 10.1093/genetics/148.1.19 PMC1459771

[88] Wippel K, Long SR 2016 Contributions of *Sinorhizobium meliloti* transcriptional regulator DksA to bacterial growth and efficient symbiosis with *Medicago sativa*. J Bacteriol 198:1374–1383. doi:10.1128/JB.00013-16. 26883825 PMC4836237

[89] Barnett MJ, Long SR 2018 Novel genes and regulators that influence production of cell surface exopolysaccharides in *Sinorhizobium meliloti*. J Bacteriol 200:e00501-17. doi:10.1128/JB.00501-17. 29158240 PMC5763050

[90] Csárdi G, Nepusz T 2006 The igraph software package for complex network research. InterJournal Complex Systems:1695 http://igraph.org.

[91] Fruchterman TM, Reingold EM 1991 Graph drawing by force‐directed placement. Softw Pract Exp 21:1129–1164. doi:10.1002/spe.4380211102.

[92] Meade HM, Long SR, Ruvkun GB, Brown SE, Ausubel FM 1982 Physical and genetic characterization of symbiotic and auxotrophic mutants of *Rhizobium meliloti* induced by transposon Tn*5* mutagenesis. J Bacteriol 149:114–122. 6274841 10.1128/jb.149.1.114-122.1982 PMC216598

[93] Finan TM, Kunkel B, De Vos GF, Signer ER 1986 Second symbiotic megaplasmid in *Rhizobium meliloti* carrying exopolysaccharide and thiamine synthesis genes. J Bacteriol 167:66–72. doi:10.1128/jb.167.1.66-72.1986. 3013840 PMC212841

