# Supplementary figures and images for: Most Sinorhizobium meliloti Extracytoplasmic Function Sigma Factors Control Accessory Functions
Source: mSphere. 2018 Oct 10;3(5):e00454-18. doi: 10.1128/mSphereDirect.00454-18 (PMC6180224; doi:10.1128/mSphereDirect.00454-18)

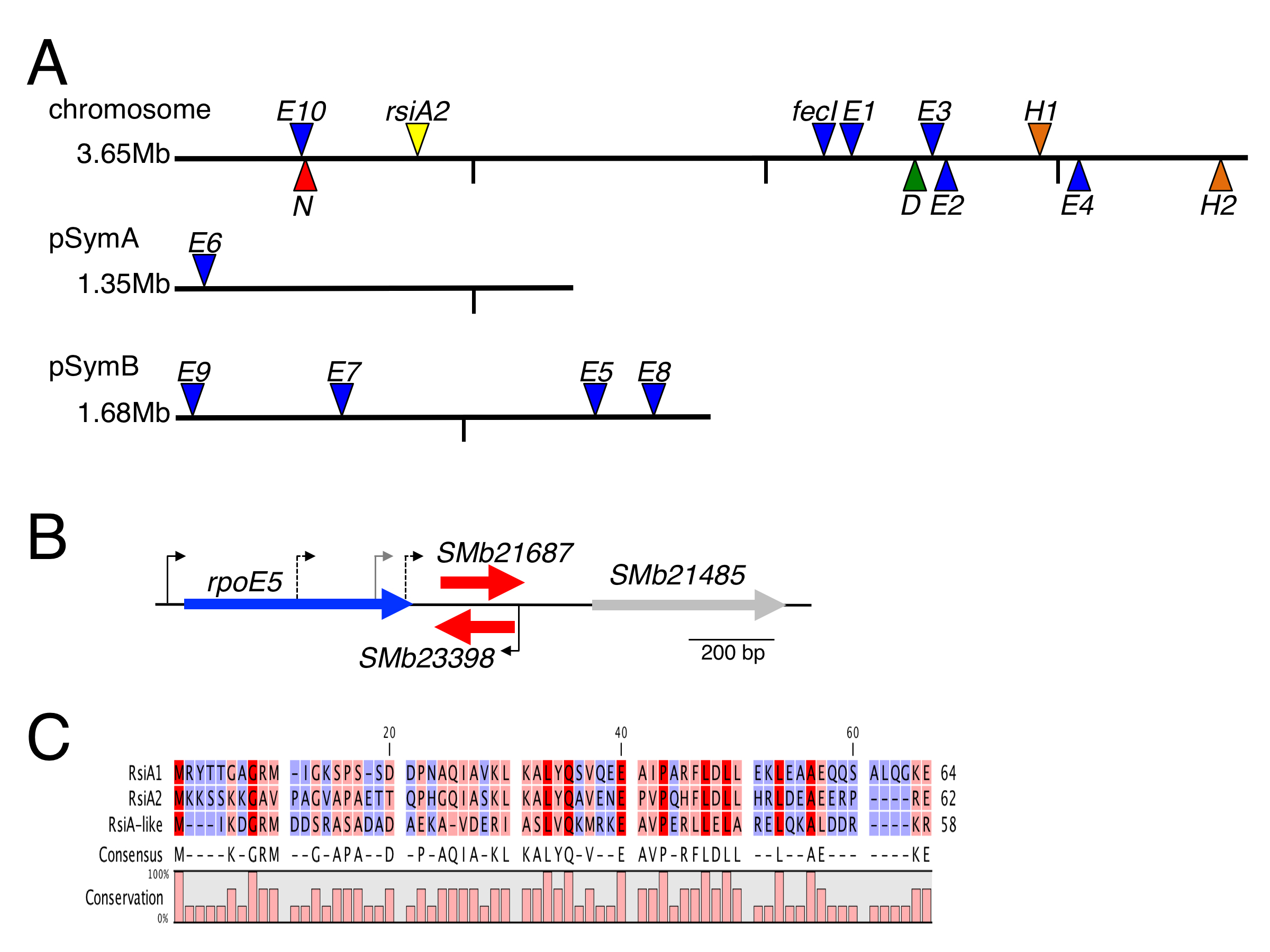

Supplement: FIG S1 [file sph005182653sf1.jpg]

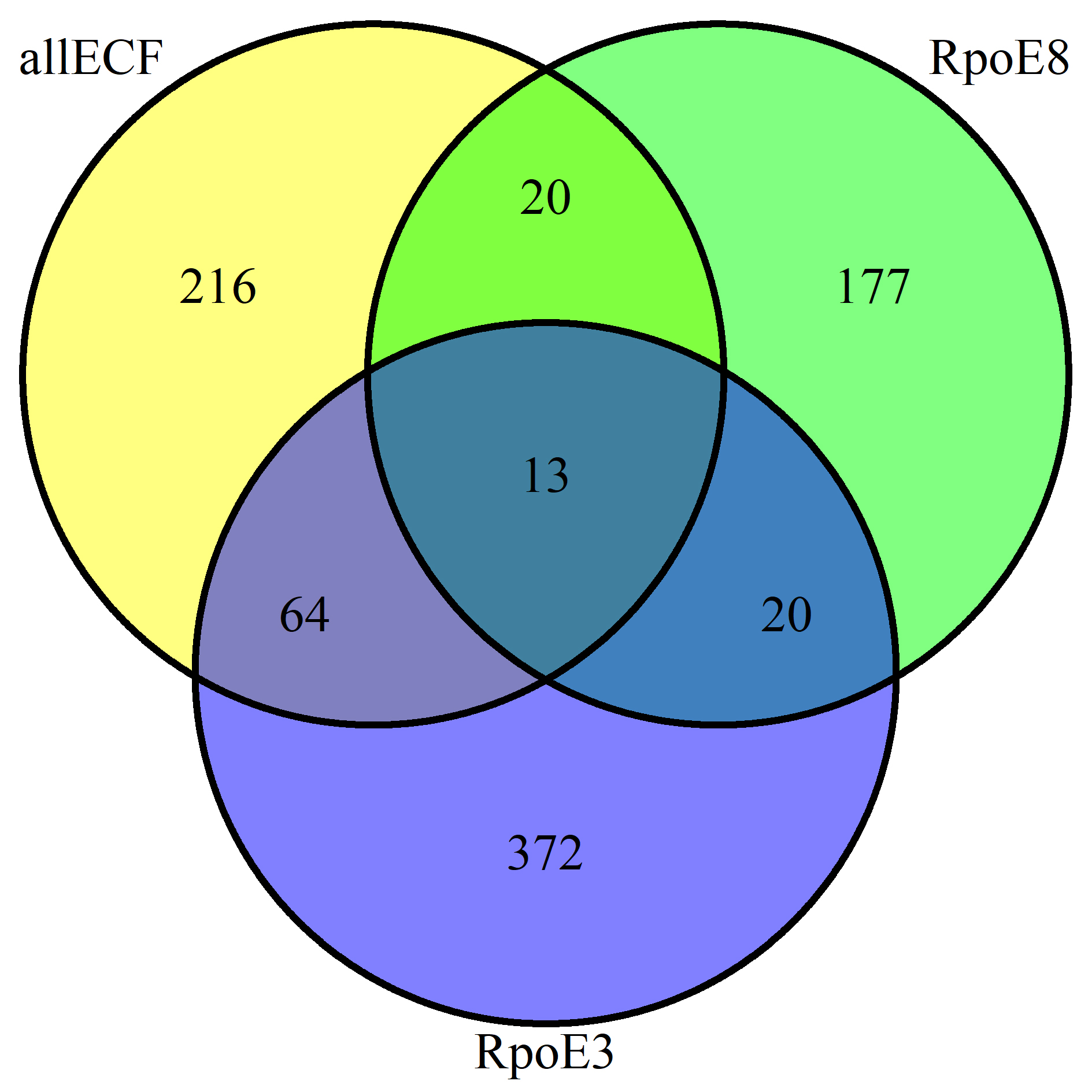

Supplement: FIG S2 [file sph005182653sf2.jpg]

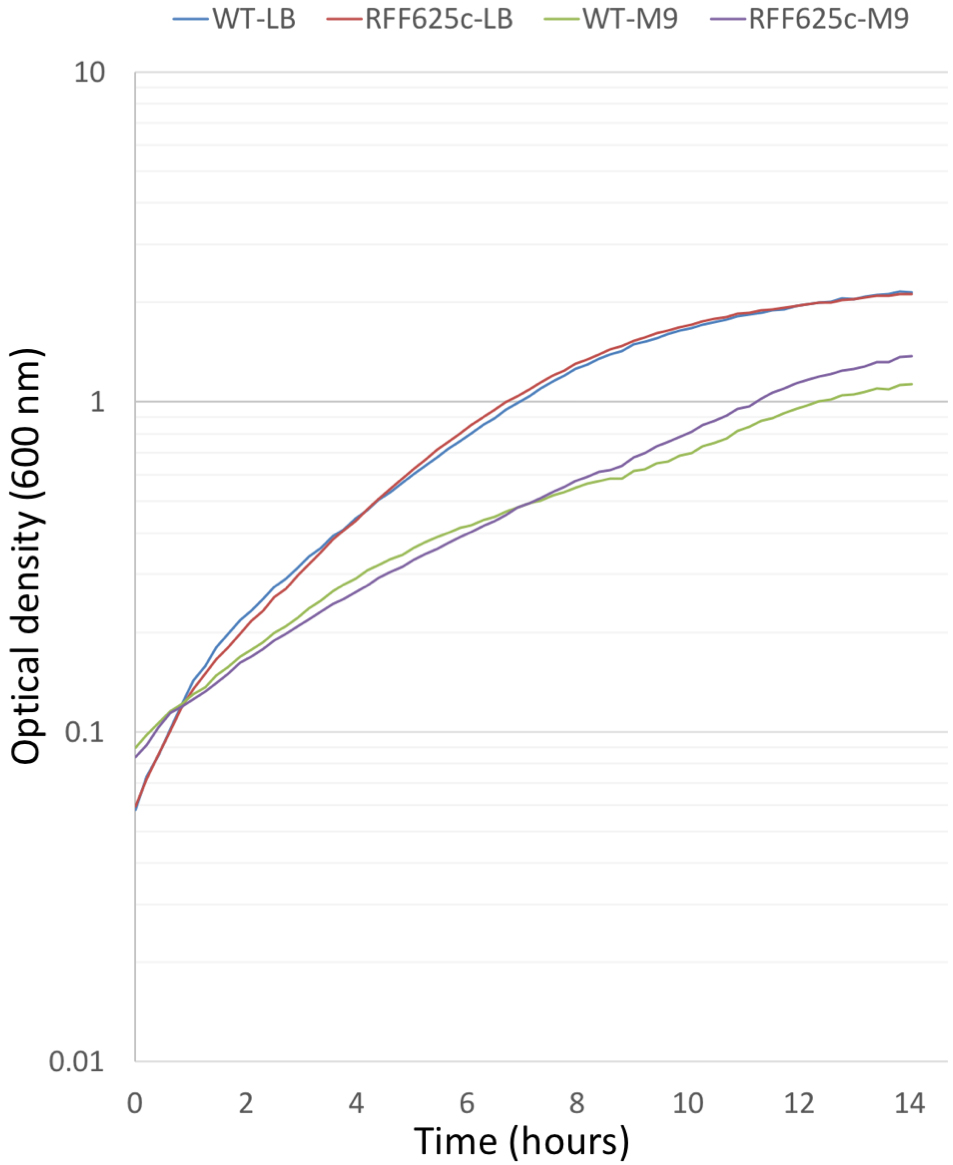

Supplement: FIG S3 [file sph005182653sf3.jpg]
