# Supplementary material for: Most Sinorhizobium meliloti Extracytoplasmic Function Sigma Factors Control Accessory Functions
Source: mSphere. 2018 Oct 10;3(5):e00454-18. doi: 10.1128/mSphereDirect.00454-18 (PMC6180224; doi:10.1128/mSphereDirect.00454-18)
Supplement: TABLE S1 [file sph005182653st1.docx]

| **ECF sigma/anti-sigma gene(s) deleted** | **SM identifier(s) in genome annotation** | **Base pairs deleted between** |
| --- | --- | --- |
| *rpoE1-ecfR1* | SMc01419-N/A | SMc 2,267,072 – 2,268,436 |
| *rpoE2-rsiA1* | SMc01506-SMc01505 | SMc 2,624,217 – 2,623,456 |
| *rpoE3-ecfR3* | SMc02713-SMc02714 | SMc 2,562,688 – 2,564,039 |
| *rpoE4-ecfR4* | SMc01451-SMc01450 | SMc 3,054,431 – 3,055,744 |
| *rpoE5-SMb21687* | SMb21484-SMb21687 | SMc 1,405,317 – 1,406,103 |
| *rpoE6-ecfR6* | SMa0143-SMa0144 | SMa 78,964 – 80,293 |
| *rpoE7-ecfR7* | SMb20531-SMb20532 | SMb 554,979 – 556,160 |
| *rpoE8-SMb20593* | SMb20592-SMb20593 | SMb 1,606,088 – 1,607,379 |
| *rpoE9-SMb20029* | SMb20030-SMb20029 | SMb 40,952 – 42,281 |
| *rpoE10-SMc01151* | SMc01150-SMc01151 | SMc 400,979 – 402,620 |
| *fecI-fecR* | SMc04203-SMc04204 | SMc 2,188,691 – 2,190,229 |
| N/A*-rsiA2* | SMc04884 | SMc 815,824 – 816,026 |

**Table S1**
