# Supplementary material for: Most Sinorhizobium meliloti Extracytoplasmic Function Sigma Factors Control Accessory Functions
Source: mSphere. 2018 Oct 10;3(5):e00454-18. doi: 10.1128/mSphereDirect.00454-18 (PMC6180224; doi:10.1128/mSphereDirect.00454-18)
Supplement: TABLE S2 [file sph005182653st2.docx]

| Replicon | Position | Type | Ref. | Variant | Location | Amino acid change |
| --- | --- | --- | --- | --- | --- | --- |
| **CL150 and RFF625c** | | | | | | |
| Chromosome | 568393 | Insertion | - | C | *pstC* | Ala185fs |
| Chromosome | 2673845 | SNV | T | C | *livH* | Ile17Val |
| Chromosome | 3652461 | SNV | G | A | Intergenic region | N/A |
| **RFF625c** | | | | | | |
| pSymA | 170209 | SNV | C | T | SMa0306 | Synonymous |
| pSymB | 80214 | SNV | G | T | SMb20071 | Ala185Asp |
| pSymB | 597868 | SNV | T | G | SMb20811 | Val27Gly |
| pSymB | 637536 | SNV | A | G | SMb21014 | Synonymous |
| Chromosome | 1198694 | SNV | G | T | Intergenic region | N/A |

**Table S2**
